# A novel uORF regulates folliculin to promote cell growth and lysosomal biogenesis during cardiac stress

**DOI:** 10.1038/s41598-025-87107-3

**Published:** 2025-01-27

**Authors:** Maja Bencun, Laura Spreyer, Etienne Boileau, Jessica Eschenbach, Norbert Frey, Christoph Dieterich, Mirko Völkers

**Affiliations:** 1 Klaus Tschira Institute for Integrative Computational Cardiology, University of Heidelberg, Heidelberg, Germany; 2 Department of Cardiology, Angiology and Pneumology, University Hospital Heidelberg, Heidelberg, Germany; 3 German Centre for Cardiovascular Research (DZHK)-Partner Site Heidelberg/Mannheim, Heidelberg, Germany

**Keywords:** Hypertrophic growth, Upstream open reading frame, Folliculin, Translation, TFEB, Lysosome, TOR signalling, Cell signalling, Mechanisms of disease

## Abstract

Pathological cardiac remodeling is a maladaptive response that leads to changes in the size, structure, and function of the heart. These changes occur due to an acute or chronic stress on the heart and involve a complex interplay of hemodynamic, neurohormonal and molecular factors. As a critical regulator of cell growth, protein synthesis and autophagy mechanistic target of rapamycin complex 1 (mTORC1) is an important mediator of pathological cardiac remodeling. The tumor suppressor folliculin (FLCN) is part of the network regulating non-canonical mTORC1 activity. FLCN activates mTORC1 by functioning as a guanosine triphosphatase activating protein (GAP). Our work has identified a regulatory upstream open reading frame (uORF) localized in the 5′UTR of the FLCN mRNA. These small genetic elements are important regulators of protein expression. They are particularly important for the regulation of stress-responsive protein synthesis. We have studied the relevance of the FLCN uORF in the regulation of FLCN translation. We show that FLCN downregulation through the uORF is linked to cardiomyocyte growth and increased lysosomal activity. In summary, we have identified uORF-mediated control of RNA translation as another layer of regulation in the complex molecular network controlling cardiomyocyte hypertrophy.

**Supplementary Information:**

The online version contains supplementary material available at 10.1038/s41598-025-87107-3.

## Introduction

Cardiovascular diseases (CVDs) are the leading cause of death worldwide^1^. The development of these conditions is often exacerbated by chronic pathological stress on the heart, leading to hypertrophic growth and an elevated level of protein synthesis. Protein synthesis is a tightly regulated process with regulation occurring at the transcriptional as well as translational level. Post-transcriptional regulation of gene expression is recognized as an important contributor to maladaptive changes of the heart^2–5^. Upstream open reading frames (uORFs) are post-transcriptional regulatory elements on mRNA that allow cells to rapidly change their translational output in response to external and internal stimuli. They are predominantly correlated with repressed translational efficiency and have been implicated in the development of several human diseases^6–8^. Research focusing on translation in cardiomyocytes have identified uORFs as important contributors to gene expression control^3,9^. Even though about 50% of mammalian mRNAs contain uORFs^10^, the extent of their regulatory function on pathological cardiac remodeling is still unknown.

Pathological cardiac remodeling progresses to heart failure (HF) due to increased intracardiac pressure, decreased cardiac output and left ventricular hypertrophy^11^. This is associated with changes in cell size and sarcomere reorganization orchestrated mainly by increased protein synthesis. At the center of this complex signaling network is the evolutionary conserved kinase, mechanistic target of rapamycin complex 1 (mTORC1)^3,4,12^. MTORC1 is a regulator of cellular metabolism promoting protein synthesis and cell growth^13–15^. MTORC1 activity is affected by numerous cues including amino acid levels and presence of growth factors. The output of the kinase varies according to cell type and signaling stimulus starting from increase in protein synthesis and mRNA transcription to regulation of autophagy.

Lysosomes have emerged as a central sensor for cellular energy turnover and mTORC1 activation. Kinase recruitment to lysosomal surfaces is regulated by the activity of heterodimeric Rag GTPase complexes: RagA/B: RagC/D^16–19^. Their activity is determined by their nucleotide binding status. Rag A/B are active in the GTP-bound state while the Rag C/D GTPases are active in the GDP-bound form^20,21^. The GTPase-activating protein (GAP) GATOR1 mediates GTP hydrolysis of Rag A/B. The Rag C/D nucleotide state is controlled by the GAP complex of Folliculin (FLCN) and Folliculin interacting proteins 1/2 (FNIP1/2)^20,22,23^. FLCN is a ubiquitously expressed, evolutionary conserved tumor suppressor gene^24^. Loss-of-function mutations cause Birt-Hogg-Dubé (BHD) syndrome, a familial cancer characterized by tumors of the hair follicle (fibrofollicullomas), kidney and lung^25,26^. FLCN and FNIP1/2 are recruited to the lysosomal membrane under starvation conditions^27,28^. When amino acids are abundantly present the RagA/B^GTP^-RagC/D^GDP^ heterodimer recruits mTORC1 to lysosomes where kinase activation occurs^16,17,29,30^.

FLCN coordinates substrate specificity to mTORC1^31,32^. A conditional Flcn KO mouse model has highlighted its importance in the homeostasis of the heart^33^. Nevertheless, its precise involvement in cardiac biology and disease association is not well established. Our previous work showed a reduction in FLCN protein levels in hypertrophic heart tissue^3^. In the present study we examined the mechanism behind this downregulation and its relevance for hypertrophic growth of cardiomyocytes.

## Results

### Folliculin (FLCN) is an upstream open reading frame (uORF)-regulated gene

**Fig. 1:**
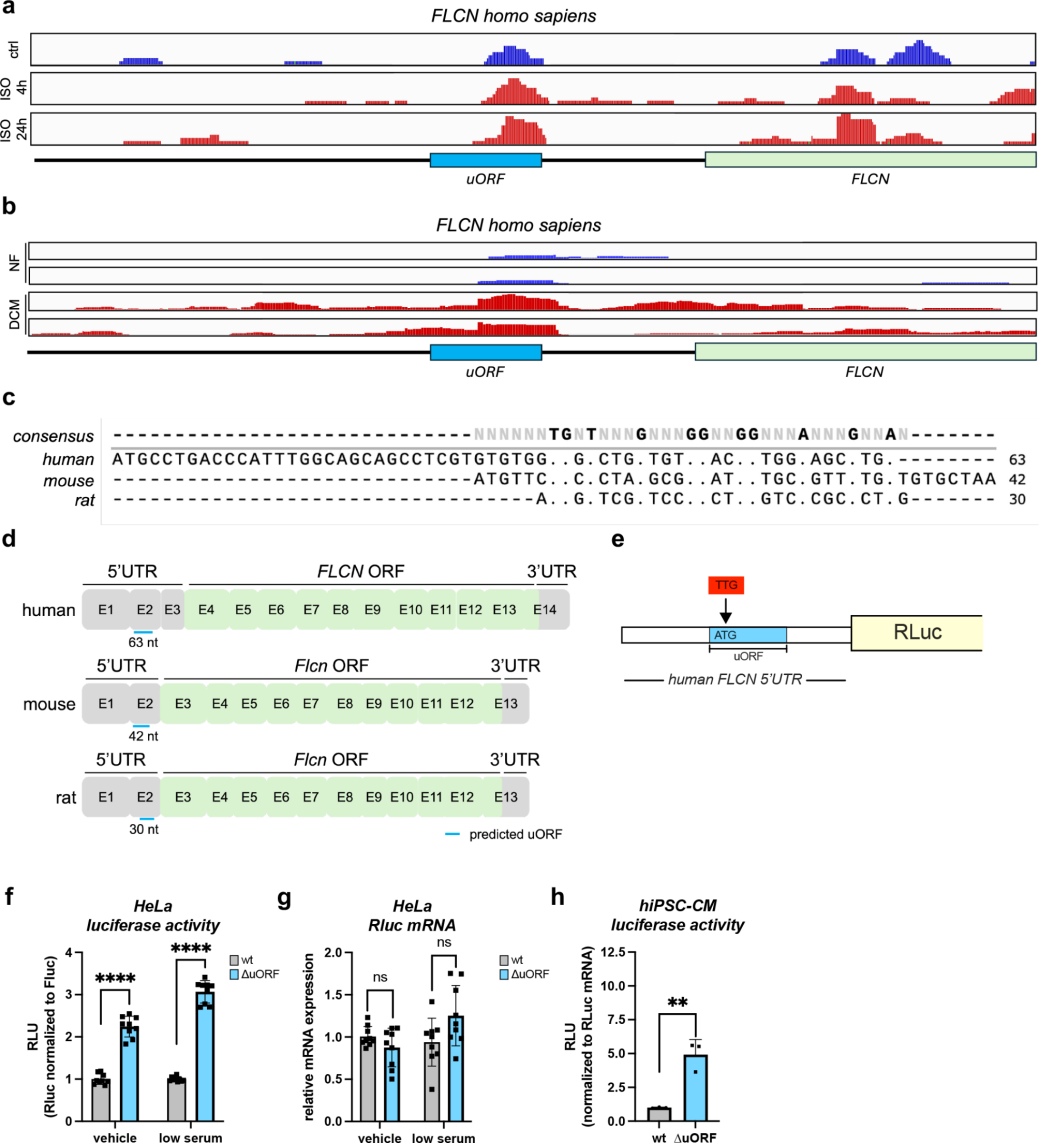
FLCN is an upstream open reading frame (uORF)-regulated gene. (**a**) Ribo-seq coverage plots in the human genomic locus containing the Folliculin (FLCN) gene show accumulation of ribosomes in the 5′UTR of the transcript in human induced pluripotent stem cell-derived cardiomyocytes (hiPSC-CMs). Ribosome occupancy of the FLCN 5′UTR (E1 + E2) and a part of the exon containing the start codon (E3) is shown. The putative uORF present in the human and murine FLCN mRNA transcripts is encoded on exon 2 (E2). (**b**) Ribo-seq coverage for FLCN in human heart samples.* NF* non-failing,* DCM* dilated cardiomyopathy. (**c**) Nucleotide sequence alignment of the human, mouse and rat Flcn uORF shows the lack of sequence conservation in the three species. Alignments were done with SnapGene using the Clustal Omega settings. Conserved nucleotides are marked by dots and highlighted in bold letters in the consensus sequence on top. (**d**) The schematic representation of the human, murine and rat Flcn transcripts highlights the positional conservation of the studied uORF. The 5′ and 3′UTRs are depicted in grey. The coding exons of the mRNA transcript are highlighted in green. The location of the putative uORF is marked with a blue line. The nucleotide (nt) length of the uORF is indicated below. (**e**) Schematic depiction of the luciferase reporter plasmids. The wild-type human FLCN 5′UTR was cloned upstream of the Renilla luciferase coding sequence (RLuc). The uORF is highlighted in blue. The uORF ATG start codon was mutated to TTG in the uORF knockout constructs (designated ∆uORF in the following figures). (**f**) The regulatory potential on translation is measured by luciferase activity under normal conditions (vehicle) and during cellular stress responses (low serum) in HeLa cells.* n = 3 replicates* Data was analyzed by unpaired* t*-test. Error bars indicate ± standard deviation; **p-value ≤ 0.01; ***p-value ≤ 0.001; ****p-value ≤ 0.0001. (**g**) The bar graph shows the relative expression levels of Renilla RNA isolated and quantified from the samples shown in f.* n = 3 replicates* RT-qPCR data was analyzed by unpaired* t*-test. Error bars indicate ± standard deviation, ns: not significant. (**h**) In vitro transcribed Rluc mRNA was transfected into hiPSC-CMs and luciferase activity was measured under standard culture conditions. Luciferase activity was normalized to Rluc mRNA levels. Data was analyzed by unpaired* t*-test. Error bars indicate ± standard deviation; **p-value ≤ 0.01

Our work on the translated transcriptome of cardiomyocytes scored FLCN as a potentially uORF-regulated gene that showed decreased translation levels during cardiac remodeling in response to pressure overload in mice^3^. The putative uORF was also identified in ribosome profiling experiments in neonatal rat cardiomyocytes^3^. The downregulation of FLCN translation efficiency is only observed during pathological cardiac remodeling in the murine heart (Supplementary Fig. 1). The putative uORF was also identified in translational profiling experiments of human induced pluripotent stem cell-derived cardiomyocytes (hiPSC-CM) and from human cardiac tissue (Fig. 1a,b). The uORF sequence is not conserved between the three studied species (Fig. 1c). However, the uORF is encoded in the 2nd exon of the FLCN gene in all three genomes arguing for positional conservation of the uORF (Fig. 1d).

To analyze the functional role of the uORF, we cloned the 5′UTR of the human FLCN gene upstream of the *Renilla* luciferase reporter (Fig. 1e). Additionally, we introduced a single nucleotide exchange mutating the ATG start codon of the uORF to a TTG (∆uORF). HeLa cells were transfected with the reporter plasmids and luciferase activity was measured under standard culture conditions (vehicle) and during cell stress (low serum) (Fig. 1f). We observed a 2–3-fold increase in luciferase activity following uORF deletion. The lack of a significant difference in mRNA expression level of *Renilla* luciferase implies that the observed difference in luciferase activity is not due to differential expression level of the two constructs but rather due to differences in regulation at the translational level (Fig. 1g).

To confirm that the uORF is also functional in cardiac myocytes we transfected human induced pluripotent stem cell-derived cardiomyocytes (hiPSC-CMs) with the 5′UTR-luciferase constructs. Cardiomyocytes are difficult to transfect with plasmids. We generated in vitro transcribed mRNA of our *Renilla* reporter genes and transfected the RNA into hiPSC-CMs. The luciferase activity increased up to 5-fold upon uORF deletion (Fig. 1h).

### FLCN protein levels can be altered by uORF-targeting antisense oligonucleotides (ASO)

**Fig. 2:**
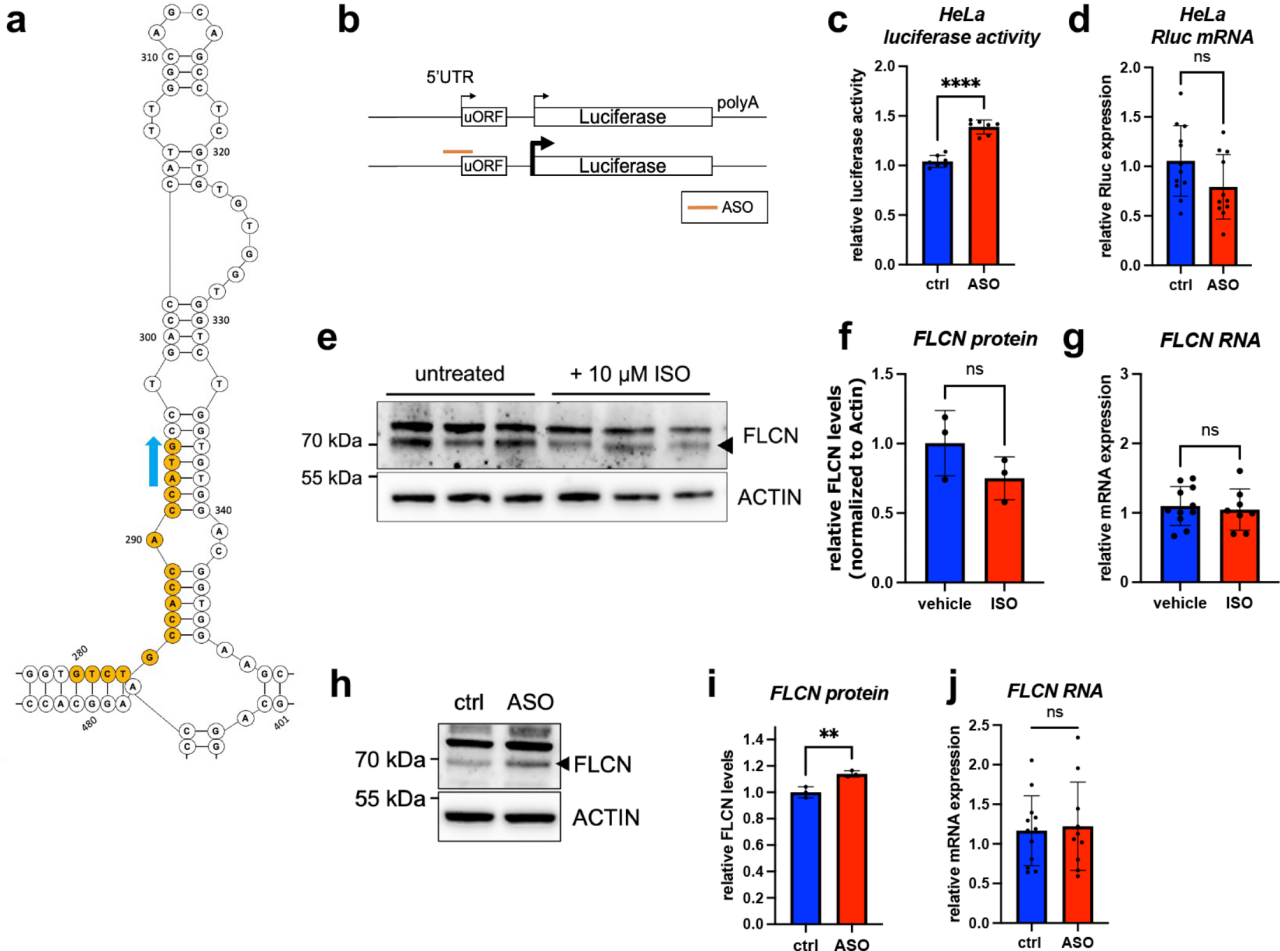
Antisense oligonucleotides targeting the FLCN uORF cause translational upregulation of FLCN protein. (**a**) The predicted secondary structure of the partial FLCN 5′UTR encoding the uORF is depicted. The start codon of the uORF is indicated by a blue arrow. Nucleotides bound by the ASO are highlighted in orange. Structure predictions were performed by RNAfold using the complete FLCN mRNA (ensemble ID: ENST00000285071.9). (**b**) An overview of the luciferase reporter experiments with ASO transfections is presented. (**c**) The Luciferase activity was measured in HeLa cells 24 h after transfection of the wild-type luciferase reporter plasmid and either a control ASO (ctrl) or FLCN uORF-targeting ASO (ASO). Data was analyzed by unpaired *t*-test. Error bars indicate ± standard deviation; **p-value ≤ 0.01; ***p-value ≤ 0.001; ****p-value ≤ 0.0001. *n = 7 replicates*. (**d**) The bar graph shows the relative expression levels of *Renilla* RNA isolated and quantified from the samples shown in (c). *n = 7 replicates* RT-qPCR data was analyzed by unpaired *t*-test. Error bars indicate ± standard deviation, *ns* not significant. (**e**) The immunoblot shows FLCN protein levels in human induced pluripotent stem cell-derived cardiomyocytes (hiPS-CM). The cells were treated with 10 µM isoprenaline (ISO) for 24 h. The black arrowhead indicates the FLCN specific protein band. ACTIN was used as loading control. (**f**) The bar graph shows the quantification of band intensities from (**e**). Band intensities were normalized to ACTIN levels. (**g**) RT-qPCR measurements were performed to quantify FLCN expression levels after ISO stimulation. *n* = *2 replicates* Data was analyzed by unpaired *t*-test. Error bars indicate ± standard deviation; ns: not significant. (**h**) FLCN protein levels in hiPS-CMs following control ASO (ctrl) and FLCN uORF-targeting ASO (ASO) transfection. (**i**) Quantification of band intensities from (**h**). ACTIN was used as loading control. *n* = *2 replicates* Data was analyzed by unpaired *t*-test. Error bars indicate ± standard deviation; **p-value ≤ 0.01. (**j**) FLCN mRNA expression levels were quantified after ASO transfection in hiPSC-CMs. *n* = *2 replicates* Data was analyzed by unpaired *t*-test. Error bars indicate ± standard deviation; *ns* not significant.

To correct the uORF-mediated translational downregulation of FLCN, we designed a chemically modified antisense-oligonucleotide (ASO) targeting the start codon of the uORF (Fig. 2a). This would inhibit the uORF-imposed decrease in translation efficiency through ASO-mediated steric inhibition of uORF translation^34^.

The ASO targeting the FLCN uORF start codon did not cause degradation of the FLCN mRNA transcript (Supplementary Fig. 2). First, we tested our uORF-targeting ASO in HeLa cells. We used the luciferase reporter plasmid encoding the wildtype 5′UTR sequence of FLCN. We co-transfected the uORF-targeting ASO (ASO) or a scrambled control (ctrl) with the reporter plasmid (Fig. 2b). The uORF-targeting ASO markedly increased luciferase activity without significantly altering luciferase RNA expression (Fig. 2c,d). To test whether the ASO could also upregulate endogenous protein levels of FLCN we first analyzed FLCN expression in hiPSC-CMs after isoprenaline (ISO) treatment. ISO stimulation led to a downregulation of FLCN at the protein but not at the RNA level (Fig. 2e–g). We then transfected the ASO in hiPSC-CMs and could confirm an upregulation of FLCN protein levels following transfection without altering mRNA expression (Fig. 2h–j). Using this approach, we could increase FLCN protein expression less dramatically than with conventional overexpression constructs (e.g. mRNA, plasmids).

### Downregulation of Flcn leads to a hypertrophic growth-like phenotype in cardiomyocytes

**Fig. 3:**
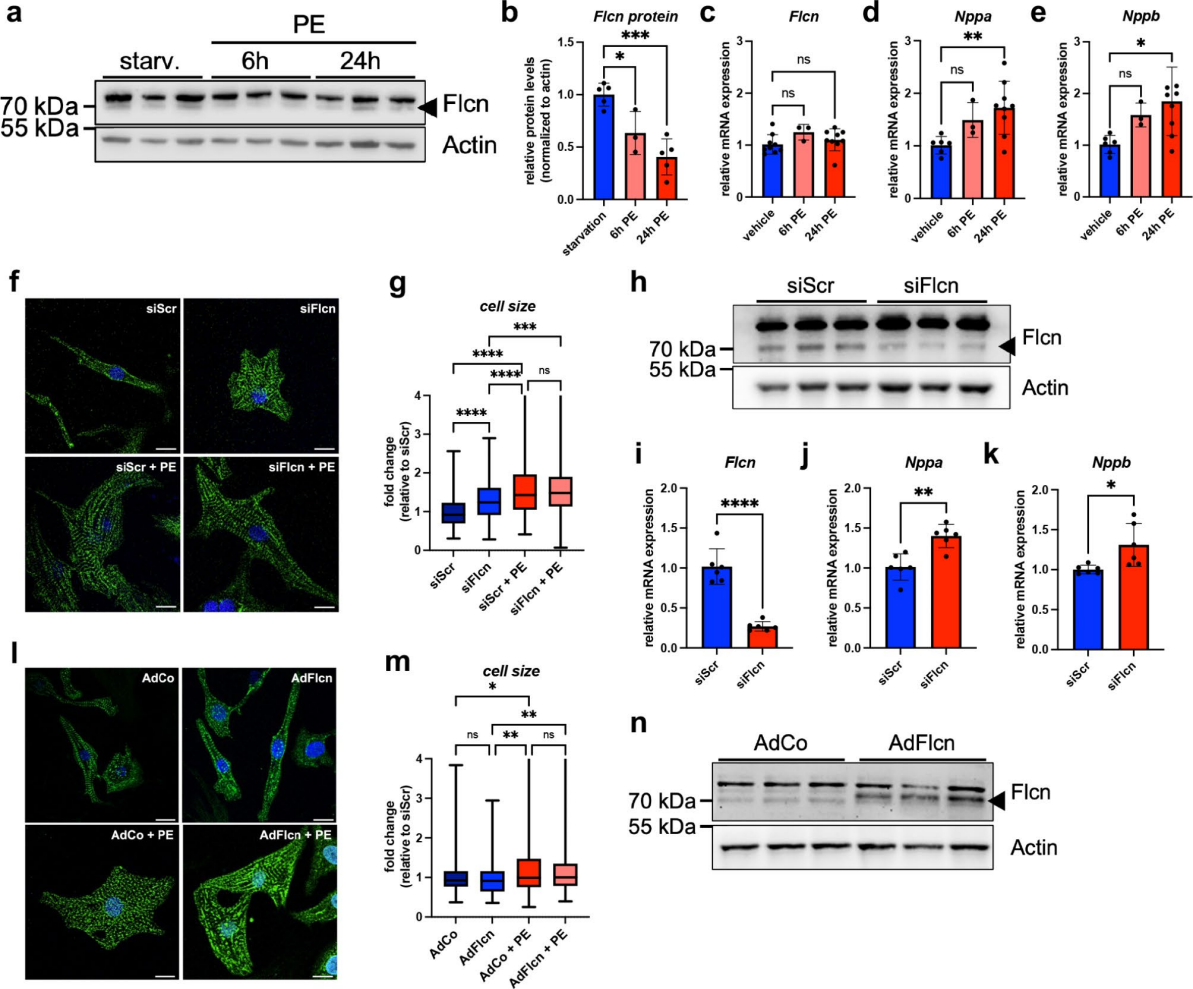
Downregulation of FLCN protein levels is required for cardiac hypertrophic growth. (**a**) Flcn protein levels in neonatal rat cardiomyocytes (NRCMs) before and after stimulation with 50 µM phenylephrine (PE) are shown. Actin was used as loading control. (**b**) The bar graph shows the quantification of Flcn band intensities from two independent experiments. Band intensities were normalized to Actin levels. (**c**–**e**) The bar graph shows the relative mRNA levels of Flcn, Nppa, and Nppb in NRCMs following PE stimulation. Data was analyzed by unpaired *t*-test. Error bars indicate ± standard deviation; *p-value ≤ 0.1; **p-value ≤ 0.01; ***p-value ≤ 0.001; ****p-value ≤ 0.0001. (**f**) Representative immunofluorescence stainings of NRCMs transfected with scrambled (siScr) or Flcn-targeting siRNA (siFlcn). The cells were stimulated with 50 µM Phenylephrine (PE) for 24 h to induce cell growth. The cells were stained for sarcomeric actinin (green) to visualize the cell size. Cell nuclei were counterstained with Dapi (blue). Scale bar represents 20 μm. (**g**) Box plots show the cell size after Flcn KD (siFlcn) normalized to scrambled siRNA transfected control (siScr). *n = 3 replicates.* (**h**) Representative immunoblot of siRNA-mediated Flcn KD in NRCMs (siFlcn). The black arrowhead indicates the Flcn specific protein band. Actin was used as loading control. The control cells were transfected with a scrambled non-targeting siRNA (siScr). (**i**) The bar graph shows the relative Flcn mRNA levels after siRNA-mediated Flcn KD. (**j** + **k**) The bar graphs show the relative mRNA levels of Nppa and Nppb in NRCMs after Flcn KD. (**l**) Representative immunofluorescence stainings of NRCMs transduced with control (AdCo) or Flcn-encoding adenoviruses (AdFlcn) under starvation conditions. The cells were stained for sarcomeric actinin (green) to visualize the cell size. Cell nuclei were counterstained with Dapi (blue). Scale bar represents 20 μm. (**m**) Box plots show the cell size after Flcn OE (AdFlcn) normalized to NRCMs transduced with control virus (AdCo). *n = 3 replicates*. (**n**) Representative immunoblot after adenoviral transduction for Flcn overexpression (OE) studies (AdFlcn). The black arrowhead indicates FLCN specific protein bands. Actin was used as loading control. Control cells were transduced with a control adenovirus (AdCo).

We further wanted to understand the significance of this downregulation during pathologic cardiac remodeling. First, we confirmed that the downregulation observed in mouse cardiac myocytes following pressure overload in vivo^3^ could be reproduced in our in vitro model system using neonatal rat cardiomyocytes (NRCMs). We used two stimulants of hypertrophic growth of in vitro-cultured NRCMs: phenylephrine (PE) stimulation (Fig. 3a) and culturing in culture media containing 10% FCS (high serum) (Supplementary Fig. 3). Both treatments led to a downregulation of Flcn at the protein but not at the RNA level (Fig. 3a–c, Supplementary Fig. 3). Nppa and Nppb mRNA levels were used as a marker for induction of cardiomyocyte remodeling (Fig. 3d,e).

Next, we knocked down Flcn by siRNA transfection in NRCMs (Fig. 3h). Following Flcn knockdown (KD) the cardiac myocytes exhibited a slight, yet significant, increase in cell size under starvation conditions (Fig. 3f,g). This difference disappeared after PE stimulation. The KD of Flcn itself led to a significant increase in Nppa and Nppb RNA transcripts (Fig. 3i–k). In parallel, we also studied the consequences of Flcn overexpression (OE) by adenoviral transduction (Fig. 3l–n). OE of the protein did not lead to observable changes in cell size (Fig. 3l,m).

### Flcn downregulation does not influence mTORC1-mediated regulation of cellular translation

**Fig. 4:**
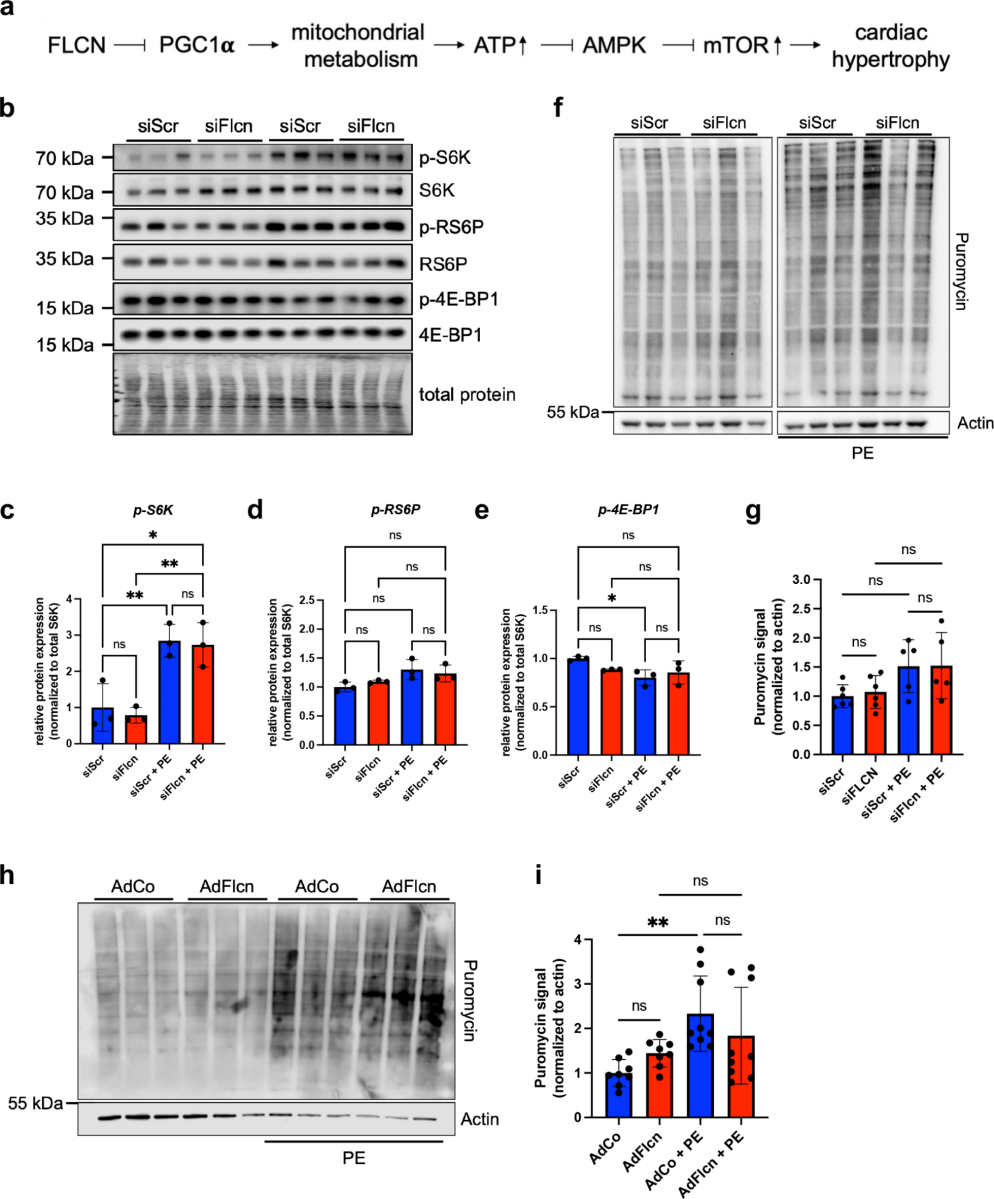
Reduced FLCN expression does not influence mTORC1-mediated regulation of cellular translation. (**a**) Schematic depiction of the signaling pathway highlighting the FLCN protein and its role in cardiac remodeling. (**b**) Representative immunoblot showing the expression and phosphorylation state of downstream mTORC1 targets in NRCMs after Flcn KD with and without PE stimulation. (**c**–**e**) Quantification of band intensities for the proteins shown in (**b**). (**f**) Representative immunoblot of the puromycin incorporation assay in NRCMs after transfection of scrambled (siScr) or Flcn-targeting (siFlcn) siRNA followed by stimulation with PE for 24 h. (**g**) Quantification of the immunoblot shown in (**f**). Puromycin incorporation was normalized to Actin band intensities. (**h**) Representative immunoblots of the puromycin incorporation assay of NRCMs after adenoviral transduction with control Adenovirus (AdCo) or Flcn-overexpressing virus (AdFlcn) followed by stimulation with PE for 24 h. (**i**) Quantification of the Immunoblots shown in (**h**). Puromycin incorporation was normalized to Actin band intensities.

Complete Flcn knockout (KO) causes upregulation of mTORC1 activity and hypertrophic growth in murine hearts^33^. According to this model, protein translation levels are increased due to the missing inhibitory function of Flcn on mTORC1 (Fig. 4a). We did see an increase in cell size after Flcn KD (Fig. 3f,g). To assess whether this was due to aberrant mTORC1 activation and consequently elevated translation levels, we analyzed the phosphorylation state of three downstream targets of canonical mTORC1 signaling (Fig. 4b–e). We did not observe significant changes in activation state in the Flcn KD samples (Fig. 4b–e). We also measured protein synthesis by assaying incorporation of the aminoacyl-tRNA analogue puromycin into newly translated proteins after 24 h of PE stimulation. We found no differences in puromycin incorporation in Flcn KD or Flcn OE NRCMs (Fig. 4f–i). We also performed this experiment using high serum stimulation and found no difference in overall translation levels (Supplementary Fig. 3).

Flcn is also implicated in the regulation of mitochondrial dynamics and metabolism^35^. We therefore assayed RNA expression of different mitochondrial markers following Flcn KD in NRCMs (Supplementary Fig. 4). Apart from mitofusin 2 (Mfn2), we did not observe significant changes in expression levels of mitochondrial markers. Mfn2 is a regulator of mitochondrial networks, regulating mitochondrial fusion and ER-mitochondria contacts^36^ and is upregulated in heart failure.

### FLCN alters lysosomal biogenesis in cardiomyocytes

**Fig. 5:**
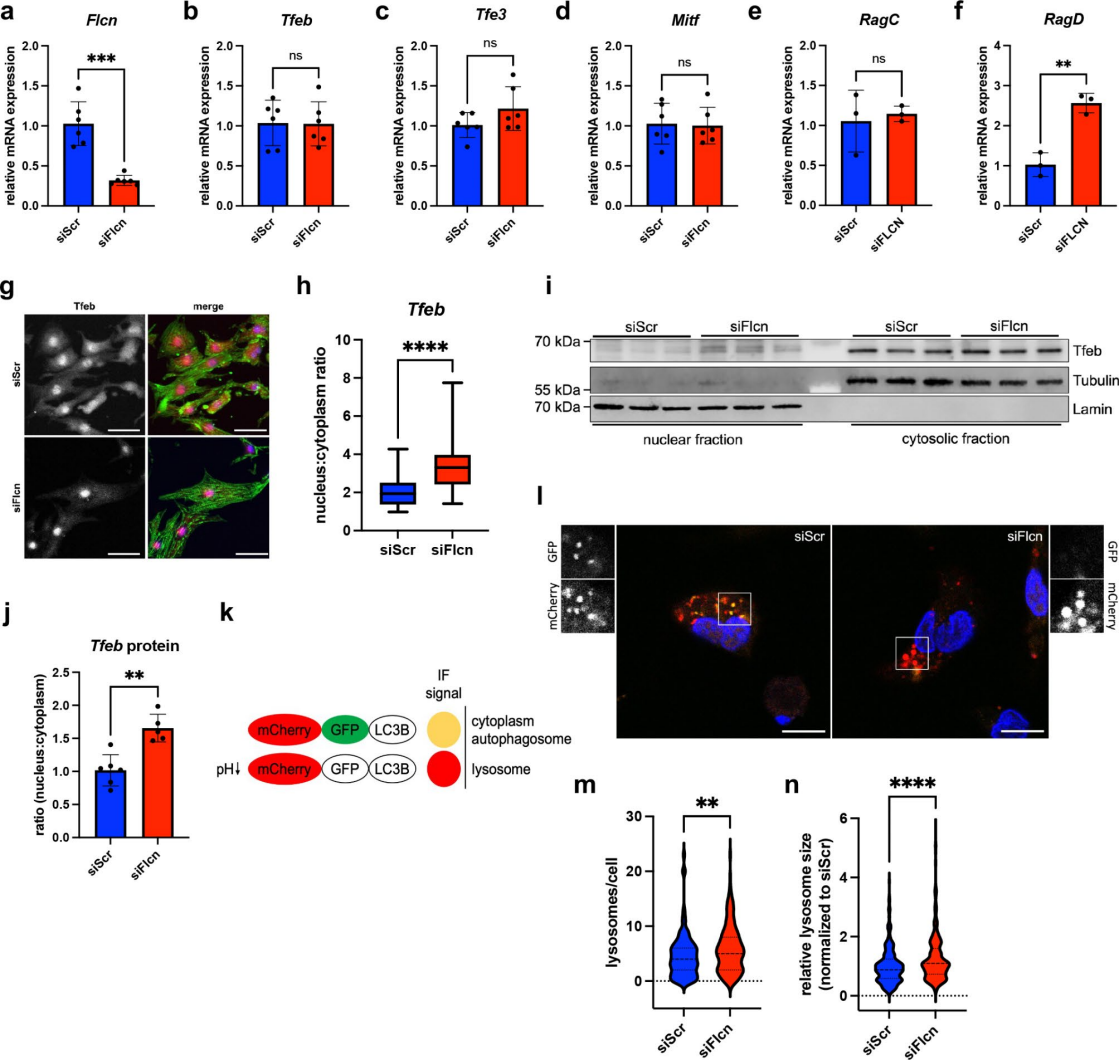
The Flcn-Tfeb signaling axis alters lysosomal biogenesis in cardiomyocytes. (**a**–**f**) The bar graphs show the relative mRNA levels in NRCMs with and without Flcn KD for Flcn, transcription factor EB (Tfeb), transcription factor binding to IGHM enhancer 3 (Tfe3), melanocyte inducing transcription factor (Mitf) and the Rag GTPases C (RagC) and D (RagD). (**g**) Representative immunofluorescence images of NRCMs stained for Tfeb following Flcn KD. Cells were stained for Tfeb (red in the merged image) and sarcomeric actinin (green). A counterstaining with Dapi was performed to visualize cell nuclei. Scale bar represents 20 μm. (**h**) The box plot depicts the ratio of nuclear to cytoplasmic signal intensities of Tfeb as quantified by ImageJ. Data was analyzed by unpaired *t*-test. ****p-value ≤ 0.0001. *n* = *2 replicates*. (**i**) Subcellular fractionation of NRCMs after Flcn KD was performed to analyze Tfeb distribution. A representative immunoblot is shown. Lamin was used as marker for the nuclear fraction and Tubulin as marker for the cytosolic fraction. (**j**) The bar graph depicts the nuclear to cytoplasm ratio of Tfeb protein band intensities as quantified by ImageJ. The nuclear Tfeb signal was normalized to Lamin band intensities and cytosolic Tfeb was normalized to Tubulin signal. Data was analyzed by unpaired *t*-test. Error bars indicate ± standard deviation; **p-value ≤ 0.01; ***p-value ≤ 0.001; ****p-value ≤ 0.0001. *n* = *2 replicates*. (**k**) Schematic depiction of the AAV6 reporter construct used to study lysosome dynamics in NRCMs. The fluorescence of GFP is quenched by low pH in lysosomes or late endosomes. The mCherry signal is retained for a longer time in lysosomes and late endosomes. (**l**) Representative immunofluorescence images of mCherry-GFP-LC3 vesicles using the AAV6 reporter outlined in (**k**). The effect of Flcn KD on lysosome dynamics in NRCMs is shown. Insets show selected fields magnified and separated into the different channels (GFP, mCherry). (**m**) Quantification of the lysosomes/cell in NRCMs following siRNA transfections to KD Flcn expression (siFlcn). A scrambled siRNA was transfected as control (siScr). Only the red dots (mCherry signal) were quantified. (**n**) Quantification of lysosome size in NRCMs transfected with a scrambled control siRNA (siScr) or a siRNA targeting Flcn (siFlcn).

Flcn suppresses the nuclear translocation of the transcription factors EB (Tfeb) and transcription factor binding to IGHM enhancer E3 (Tfe3)^31^. Tfeb and Tfe3 are master regulators of autophagy, lysosomal biogenesis and lipid metabolism regulating the coordinated lysosomal expression and regulation (CLEAR) network^37–41^. These transcription factors bind to the CLEAR sequences of target genes and thereby activate lysosomal gene expression. We analyzed the RNA levels of all the MiTF-family of transcription factors and found no significant difference in transcription levels following Flcn KD (Fig. 5a–d). Tfec was not detected in our samples.

To assess the transcriptional activity of the MiTF-family of transcription factors we profiled a panel of lysosomal marker genes by RT-qPCR (Supplementary Fig. 5). For most of the profiled genes, we did not observe a significant increase in expression levels.

We also assessed expression levels of the Rag GTPases RagC and RagD (Fig. 5e,f). These GTPases are known targets of Tfeb^42^. We found a significant upregulation of Rag D GTPase transcripts following Flcn KD (Fig. 5f). There was no significant change in Rag C expression (Fig. 5e).

Finally, we quantified the levels of glycoprotein nonmetastatic melanoma B (Gpnmb) (Supplementary Fig. 6). Gpnmb is a MiTF-regulated gene and is used as a readout for MiTF transcriptional activity^43–45^. KD of Flcn with subsequent high serum stimulation led to a significant increase in Gpnmb expression indicating elevated transcriptional activity of the MiTF transcription factors. On the other hand, OE of Flcn with subsequent stimulation inhibited Gpnmb expression in NRCMs (Supplementary Fig. 5).

MiTF transcription factors are negatively regulated by mTORC1-mediated phosphorylation. Phosphorylation mediates cytoplasmic localization and inhibits nuclear translocation of the transcription factors. Flcn mediates this regulation by recruiting the transcription factors to the lysosomal membrane and enabling interaction with mTORC1^27,46,47^. To confirm the effect of Flcn KD on MiTF transcription factors we studied changes in the subcellular localization of Tfeb following Flcn KD by immunofluorescence (IF) staining and immunoblot (Fig. 5g–j). We found a significant increase in nuclear localization of Tfeb in IF stainings after Flcn KD under physiological conditions (Fig. 5g,h). We could also confirm increased translocation of Tfeb into the nucleus upon Flcn KD by immunoblot (Fig. 5i,j).

To further investigate the effect of Flcn KD on autophagosome fusion with lysosomes we used adeno-associated virus 6 (AAV6) encoding a reporter construct with mCherry and GFP genes fused in tandem to LC3B (Fig. 5k). Lysosomes in contrast to autophagosomes have an acidic internal pH which allows lysosomal hydrolases to become active^48,49^. While mCherry is stable at an acidic pH, GFP is not, and is quickly quenched in the acidic environment of lysosomes. We transduced NRCMs with these constructs under starvation conditions and quantified the mCherry-positive lysosomal structures per cell (Fig. 5l–n). Upon Flcn KD NRCMs exhibited slightly, yet significantly, more acidic compartments per cell than the scrambled control (Fig. 5m). Additionally, we measured the average size of these lysosomal structures (Fig. 5n). On average the Flcn KD exhibited slightly bigger lysosomes.

In conclusion, this data provides evidence that Flcn downregulation during cardiac remodeling primarily serves to enhance Tfeb translocation into the nucleus thereby altering lysosomal structures in the cells.

## Discussion

Altered protein expression causes a vast array of diseases many of which are caused by reduced levels of critical proteins^50^. Here, we explore the regulatory potential of an uORF for FLCN, an important gene that has become the focus of non-canonical mTORC1 signaling^51^.

We show that the tumor suppressor FLCN is regulated through uORF-mediated translational control. The precise mechanisms of how the 5′UTR regulates translation is still incompletely understood. However, the functional relevance of uORFs in the translation process is well described^52–54^. Many uORFs show little sequence conservation but strong positional conservation within a transcript^9,55^. This regulation seems to come into play during pathological hypertrophic growth^3^. Though the uORF identified in the FLCN 5′UTR shows no sequence conservation in the three organisms studied, there is clear evidence for positional conservation of this regulatory element. We could show that the gene is downregulated at the protein but not at the mRNA level with multiple chemical stimuli and in two different in vitro model systems. Mechanistically, we show that this regulation can be perturbed using chemically modified antisense oligonucleotides. We used ASOs to increase FLCN protein levels in hiPS-CMs. ASO-mediated upregulation of protein levels was modest. However, our studies have shown that FLCN protein levels are also only slightly downregulated following hypertrophic growth remodeling, arguing for the use of ASOs to modify expression levels of deregulated proteins.

As previously noted, FLCN protein levels are only slightly downregulated in animals suffering from cardiac pressure overload^3^. Our experiments indicate that this downregulation is linked to cardiac hypertrophic growth. However, we only observed an effect on hypertrophic growth upon protein KD. The overexpression of FLCN could not prevent hypertrophic growth upon PE stimulation. To exert its function as a Rag GTPase FLCN assembles into a heterodimer with FNIP1/2. Artificially increasing FLCN by adenoviral transduction of overexpression vectors leads to an increase in the protein itself, but not necessarily of its interaction partners. Without its interaction partners the overexpressed FLCN will not be present in a functional state in the cells^56^.

Furthermore, we show that FLCN does not signal directly through the canonical mTOR signaling axis and that there are two separate mTOR-dependent pathways at play: FLCN–TFE3/TFEB and the canonical TSC1/2–mTORC1–S6K/4E-BP1 axis.

The transcription factor gene family MiTF is a highly conserved group of four transcription factors: MiTF, TFE3, TFEB and TFEC. TFE3 and TFEB are broadly expressed while the other two display a more restricted tissue expression in melanocytes and monocytes^57–59^. The transcription factors TFEB and TFE3 activate genes that regulate lysosomal biogenesis, as well as autophagy, lipid metabolism and innate immune signaling^37,39–41,60^. Lysosomes are upregulated during acute cell stress, such as starvation, oxidative stress, or cell damage. Lysosomal protein degradation is an important tool for cells to recycle their products to produce energy and promote cell survival. The slight increase in lysosomes per cell in FLCN KD cells correlates well with the observed slightly altered expression of lysosomal genes.

In our experimental system we used siRNA-mediated KD of FLCN expression, which causes only a short-term reduction in protein levels. Yet, TFEB and TFE3 together with mTORC1 and FLCN act in a feedback loop in which mTORC1 inhibits transcription factor function and the transcription factors positively regulate mTORC1 activity through transcriptional activation of Rag C/D^42^. It is conceivable that a longer-term KD of FLCN would lead to a more pronounced phenotype concerning lysosomal gene expression changes. The experiments performed here indicate that there are differences in autophagosome-lysosome fusion after FLCN KD. However, further experiments are needed to fully understand the effect of FLCN deregulation on lysosome biogenesis.

Furthermore, once TFE3 and TFEB are activated and translocate into the nucleus they upregulate the expression of Rag C/D GTPases. In the absence of FLCN the transcriptional activation of Rag C/D leads to an increase in RagA/B^GTP^:RagC/D^GTP^ formation which in turn leads to increased phosphorylation of the canonical mTORC1 targets S6K and 4E-BP1^51^. Rag C/D nucleotide status is not important for the canonical mTORC1 signaling^27,32^. Canonical mTORC1 signaling can occur in the absence of GTP hydrolysis. However, the FLCN: FNIP1/2-mediated regulation of Rag C/D nucleotide state is important for the regulation of the phosphorylation status of MiTF transcription factors and the induction of their transcriptional programs^21,32,61^.

This explains why a complete FLCN KO in cardiac tissue leads to hyperactivation of mTORC1 and causes a strong hypertrophic growth phenotype in mice while a KD does not^33^. Several other studies on FLCN have confirmed that the deletion of the protein in vivo is associated with increased mTORC1 activation^33,35,62–64^. In these cases, FLCN depletion led to activation of AMPK inducing autophagy and metabolic rewiring^65,66^. However, several in vitro model systems show reduced mTORC1 activity upon FLCN KD^67–69^. We did not observe changes in global protein translation levels, a cellular process regulated by mTORC1. It further delineates the importance of distinguishing between complete removal of a target gene from a biological system or working with partial removal through KD studies to answer important research questions.

Dysregulation of mTORC1 signaling is associated with various cardiovascular diseases, including cardiac hypertrophy, heart failure and arrhythmias^12,70^. Pharmacological inhibition of the signaling pathway has proven cardioprotective^4^. So far, these studies have only taken the canonical signaling axis with S6K and 4E-BP1 as downstream targets into consideration. Additional work is necessary to delineate the relevance of non-canonical mTORC1 signaling and to understand how these signals are integrated during cardiac remodeling. Since FLCN is part of this mTOR signaling cascade, its influence on cardiac function and cardiovascular diseases will need to be studied further.

Taken together our work sheds light on the effects small regulatory entities such as uORFs can have on protein expression. It further highlights the importance of understanding the non-canonical mTORC1 pathway to further our efforts to develop novel therapeutic strategies for the treatment of cardiovascular diseases.

## Materials and methods

### Isolation and culture of neonatal rat cardiomyocytes (NRCMs)

Neonatal rat cardiomyocytes were isolated from Wistar neonatal rat hearts by Percoll gradient separation as previously described^71^. Isolated NRCMs were plated on gelatine-coated plastic culture plates in DMEM-F12 medium (Thermo Fisher Scientific) supplemented with 10% FCS (Gibco, Waltham, MA, USA). The medium was changed to 0.5%-serum-containing DMEM-F12 the following day. To study the molecular mechanisms of cardiomyocyte remodeling through hypertrophy NRCMs were stimulated with 50 µM phenylephrine (Sigma-Aldrich) or DMEM-F12 supplemented with 10% FCS (high serum condition).

### Culturing cell lines

HeLa cells (ACC57, purchased from dmsz.de) and HEK293T cells were cultured in complete medium (DMEM + GlutaMAX (Gibco) + 10%FCS (Gibco), penicillin/streptomycin) in a 37 °C humidified incubator in the presence of 5%CO_2_.

### Human induced pluripotent-stem cell differentiation into cardiomyocytes (hiPSC-CMs) and culturing

The WT1.4 induced pluripotent stem cells (iPSCs) used in this study were a kind gift from Shirin Doroudgar. All cells were maintained in a 37 °C humidified incubator with 5% CO_2_. IPSCs were seeded on Matrigel (BD Bioscience, San Jose, CA, USA)-coated 6-well plates and grown to 95% confluency in Stem-MACS iPS Brew XF medium (Miltenyi Biotec, Bergisch Gladbach, Germany) containing Stem MACS iPS Brew XF Supplement (Miltenyi Biotec, Bergisch Gladbach, Germany). Once the iPSCs reached 95% confluency differentiation was started by switching the culture medium to RPMI1640/HEPES/GlutaMAX (Thermo Fisher Scientific, Waltham, MA, USA) + B27 Supplement minus Insulin (Thermo Fisher Scientific, Waltham, MA, USA) + 4 µM CHIR99021 (Merck Millipore, Burlington, MA, USA). Precisely 24 h later 3 ml/well of fresh differentiation medium was layered onto the cells (without CHIR99021). Three days post differentiation start the medium was replaced with fresh RPMI1640/HEPES/GlutaMAX + B27 Supplement minus Insulin medium supplemented with 2.5 µM IWP2 (Merck Millipore, Burlington, MA, USA). On day 5 post differentiation start the cells were switched to fresh RPMI1640/HEPES/GlutaMAX + B27 Supplement minus Insulin medium. On day 7 the media was changed to RPMI1640/HEPES/GlutaMAX + B27 Supplement (Thermo Fisher Scientific, Waltham, MA, USA) and was renewed every 2 days until day 10 when spontaneous beating could be observed throughout the culture. The cells underwent two rounds of metabolic selection by culturing them in lactate selection medium (RPMI1640 without Glucose (Thermo Fisher Scientific, Waltham, MA, USA) + 0.5 mg/ml human recombinant Albumin (Sigma-Aldrich Chemie GmbH, Taufkirchen, Germany), 0.2 mg/ml L-Ascorbic Acid 2-Phosphate (Sigma-Aldrich Chemie GmbH, Taufkirchen, Germany), and 4 mM Sodium DL-lactate solution (Sigma-Aldrich Chemie GmbH, Taufkirchen, Germany)). Cells were cultured in this selection medium for three days before switching back to cardio culture medium (RPMI 1640 with Glucose, GlutaMax, HEPES, and B27 supplement).

### HiPSC-CM β-adrenoreceptor stimulation

HiPS-CMs were detached using 0.25% Trypsin/EDTA and seeded on 12-well plates. Cells were allowed to recover for at least 48 h after plating and were stimulated with 10 µM isoprenaline (Sigma-Aldrich Chemie GmbH, Taufkirchen, Germany) for 24 h. The following day the cells ware washed once in 1× PBS and lysed in 100 µl/well of 1× RIPA buffer (1% v/v phosphatase inhibitor and protease inhibitor (Roche)) for western blotting.

### siRNA transfection

Pre-designed siRNAs were purchased from Thermo Fisher Scientific. Scrambled siRNA (SilencerSelect™ Negative Control No 1) and Flcn-targeting siRNA (siRNA ID: 196986). NRCMs were transfected using HiPerfect transfection reagent (Quiagen, Hilden, Germany) according to the manufacturer’s instructions at a final concentration of 25 nM.

siRNA transfections were performed in NRCMs cultured in 0.5% FCS. 48 h post transfection the cells were stimulated with 50 µM phenylephrine (Sigma-Aldrich) for 24 h. Following stimulation, the cells were washed with 1× PBS and either lysed in RNA lysis buffer (Zymo Research) for RNA isolation or in 1× RIPA buffer (1% v/v phosphatase inhibitor and protease inhibitor (Roche) for western blotting.

### RNA extraction, reverse transcription and real time quantitative PCR (RT-qPCR)

RNA extractions from cells were performed using the Quick-RNA™ MiniPrep kit from Zymo Research according to the manufacturer’s instructions. 100–500 ng of total RNA was reverse transcribed using the iScript™ Reverse Transcription Supermix (Bio-Rad Laboratories) according to the manufacturer’s instructions. Following RT the cDNA was diluted at a ratio of 1:10 and 2 µl were used for RT-qPCR analysis with iTAQ™ SYBR Green PCR Kit (Bio-Rad Laboratories) following manufacturer’s instructions. Ct values were normalized to HPRT and 18 S using the geometric mean of the housekeeper genes. Relative mRNA expression levels were calculated using the comparative Ct^72^ method 2^−∆∆Ct^. Primers used for RT-qPCR measurements are listed in Supplementary Table 1. Primer sequences for Gpnmb, RagC and RagD were taken from Li et al.^73^.

### Cloning FLCN overexpression vectors

The FLCN ORF was amplified from cDNA isolated from induced pluripotent stem cell using primers containing truncated attb1 and attb2 sequences used for Gateway cloning. We used the Phusion polymerase (NEB) for all amplifications. The primer sequences were as follows:

5′-ACCATGGATTACAAGGATGACGATGACAAGAATGCCATCGTGGCTC-3′ and 5′-AGAAAGCTGGGTTCAGTTCCGAGACTCCGAG-3′.

After ORF amplification a second set of primers was used to add the complete attb1 and attb2 sites for Gateway cloning. The primers were as follows:

- 5′-GGGGACAAGTTTGTACAAAAAAGCAGGCTAT-3′.
- 5′-GGGGACCACTTTGTACAAGAAAGCTGGGTT-3′.

A two-step PCR was performed for final amplification. The cycling conditions were as follows: 2 min–94 °C–5 × (15 s–94 °C–30 s–45 °C–2 min–68 °C)–20 × (15 s–94 °C–30 s–55 °C–2 min– 8 °C)–10 min 68 °C.

The PCR product was gel purified and cloned into the pcDNA3.1(+) and pAd/CMV vectors using the Gateway cloning kit (Thermo Fisher Scientific) according to the manufacturer’s instructions.

### Adenovirus production and transduction

The pAd/CMV/FLCN vector was digested with Pac I for 3 h. The restriction enzyme was inactivated for 15 min at 65 °C. 10 µg of linearized plasmid was transfected into HEK293T cells plated on 10 cm dishes using Lipofectamine™ 2000 (Thermo Fisher Scientific). Medium was refreshed the next day and cells were monitored for confluency daily. Once the cells reached 70% confluency they were split 1:3 and were monitored for cell dissociation daily. Dissociated cells were harvested by centrifugation and adenoviruses were released through three consecutive freeze-thaw cycles.

For adenoviral overexpression studies isolated NRCMs were transduced with indicated viral supernatants for 48 h.

### AAV production and transduction

The pDEST-CMV mCherry-GFP-LC3B WT plasmid was a gift from Robin Ketteler (Addgene plasmid #123230)^74^. The mCherry-GFP-LC3B construct was subcloned into the pTRUF-MLC800 AAV vector. Preparation of recombinant AAV6 in HEK283A cells was performed as previously described^5^. Punctae of mCherry/GFP signal were quantified as described by Agrotis et al.^74^.

### Immunofluorescence staining for cell size determination

For IF analysis cells were plated on 4-well chamber slides (Thermo Fisher Scientific). Cells were washed with 1× PBS twice and fixed with 4% PFA for 10 min. After fixation the cells were washed again two times with 1× PBS and permeabilized with 0.3% Triton™ X-100/PBS. The samples were then blocked with 10% horse serum for 30 min. Primary antibody was diluted in 10% horse serum/PBS/0.1% Triton™ X-100 (sarcomeric alpha actinin (Sigma-Aldrich) 1:100). Primary antibody incubation was performed overnight at 4 °C in a humidified chamber. The next day slides ware washed 3× with 1× PBS and incubated with secondary antibody for 1 h at room temperature (FITC AffiniPure Donkey Anti-Mouse IgG (Jackson ImmunoResearch) (1:100)). Nuclei were stained with DAPI (1:1000 in 1× PBS) and antifade mounting medium (VECTASHIELD) was used to mount the cover glass. Slides were analyzed by confocal microscopy (Leica SP8).

Cell surface areas were determined using ImageJ software. For each experiment at least 50 cells were measured/experimental condition.

### Dual-luciferase reporter assays in HeLa cells

The dual luciferase 5′UTR reporter plasmids were ordered from BioCat GmbH, Heidelberg, Germany as custom synthesized constructs using psiCHECK2.0 as the plasmid backbone. The ordered 5′UTR sequences are listed in Supplementary Table 2.

For dual-luciferase reporter assays HeLa cells were seeded on 12-well plates at 50% confluency. The next day 500 ng of the luciferase 5′UTR reporter plasmids were transfected using Viafect (Promega, Madison, WI, USA) according to the manufacturer’s instructions. The following day cells were lysed, and luciferase activity measurements were performed using the Dual Luciferase Reporter Assay kit (Promega, Madison, WI, USA) according to the manufacturer’s instructions. *Renilla* luciferase activity was normalized to *Firefly* luciferase expression.

### Luciferase reporter assays in hiPSC-CMs

The 5′UTR reporter plasmids used for luciferase assays in HeLa cells were linearized using the restriction enzyme Pme I (New England Biolabs, Ipswich, USA). Restriction digest was terminated by adding 1/20th volume 0.5 M EDTA, 1/10th volume 3 M NaOAc and precipitated with 2 volumes of ethanol. The linearized plasmid was used as template for in vitro transcription with mESSAGE mACHINE T7 Ultra (Thermo Fisher Scientific, Waltham, MA, USA) per manufacturer’s instructions. For the luciferase reporter assay 3.5 × 10^5^ hiPSC-CMs/well were seeded in Matrigel-coated 12-well plates. Cells were cultured for a week after seeding and were transfected with 1500 µg of in vitro transcribed luciferse mRNA. The following day cells were lysed, and luciferase activity measurements were performed using the Dual Luciferase Reporter Assay kit (Promega, Madison, WI, USA) according to the manufacturer’s instructions. After the measurements the remainder of the lysates was used to isolate RNA for RT-qPCR measurement of *Renilla* transcripts as described under Sect. 5.7. The qPCR results were used to normalize the reads of the luciferase assays.

### Antisense oligonucleotide design, ordering and transfection

The FLCN uORF-targeting antisense oligonucleotide was designed as outlined by Liang et al.^34^ and purchased from Integrated DNA Technologies, Inc. The ordered sequence was: mCmAmUmGmGmUmGmGmUmGmGmCmAmGmAmC. The lower-case m after each base indicates a phosphorothioate backbone. The sequence of the ASO used as a control (#759704) was taken from Liang et al.^75^.

HeLa cells were seeded at 40% confluency on 12-well plates. The next day the cells were transfected using RNAiMAX (Life Technologies, Thermo Fisher Scientific, Waltham, MA, USA) according to the manufacturer’s instructions. 24 h later the cells ware harvested in 100 µl 1× RIPA buffer for Western blotting or processed according to the manufacturer’s instructions for dual-luciferase reporter measurements (Promega, Madison, WI, USA).

For ASO transfections in hiPSC-CMs, the cells were washed briefly with Versene (Life Technologies, Thermo Fisher Scientific, Waltham, MA, USA), detached using 0.25% Trypsin-EDTA solution, washed once in splitting medium (RPMI 1640 with HEPES and GlutaMax, B27 supplement, 20% FCS (Thermo Fisher Scientific, Waltham, MA, USA), 2 µM Thiazovivin (Merck Millipore, Burlington, MA, USA) and seeded at 300,000 cells/well on 12-well plates. The next day, the cells received fresh cardio culture medium (RPMI 1640 with Glucose, GlutaMax, HEPES, and B27 supplement) and were allowed to recover for 48 h. The cells were transfected with 2 µM ASOs using RNAiMAX (Life Technologies, Thermo Fisher Scientific, Waltham, MA, USA) according to the manufacturer’s instructions. As with the HeLa cells, hiPSC-CMs were harvested the following day in 100 µl 1×RIPA buffer for Western blotting.

### Puromycin incorporation assay

NRCMs were cultured as described above. Cells were incubated with 0.5 µg/ml Puromycin (Merck-Millipore) for 30 min. Then, NRCMs were washed twice with ice cold PBS and lysed in 1× RIPA buffer. Following cell lysis, the samples were processed as described in the section western blot.

### Western blot

Cells were lysed on ice in 1× RIPA buffer (150 mN NaCl, 50 mM Tris, pH: 7.6, 1% Triton-X-100, 0.5% sodium deoxycholate, 0.1% SDS, 1× protease inhibitor (Complete EDTA-free protease inhibitor cocktail, Roche), 1× phosphatase inhibitor (Roche). The samples were then centrifuged for 15 min at 4 °C to remove insoluble debris. Protein concentrations were determined by DC™ Protein Assay Kit (Bio-Rad) according to manufacturer’s instructions. 5–20 µg of protein was loaded onto 4–12% Bis-Tris gradient gels (Bio-Rad). Gels were run in 1× MOPS buffer (Bio-Rad). Following SDS-PAGE proteins were transferred to PVDF membranes (Immobilon-P, 0.45 μm, Merck-Millipore). Membranes were blocked for an hour using 5% milk in TBS-T buffer and then incubated in primary antibody over night at 4 °C. Membranes were washed 3× for 10 min with TBS-T before incubation with HRP-conjugated secondary antibody diluted in 3% milk/TBS-T for 1 h. The blots were imaged using ECL solutions (BioZym).

Antibodies used in this study were: beta-ACTIN (C4) (1:5000) (Santa Cruz Biotechnology), FLCN (1:1000) (Proteintech Group), Puromycin (MABE343) (1:20000) (Merck-Millipore).

Signal quantification was done using FIJI/ImageJ. The unprocessed Western blot images was used to draw a box around the band of interest. Following background subtraction, a histogram was plotted and the area of the signal of each band was measured. The band intensities were divided by the corresponding signal of the loading control (ACTIN) from the same gel lane.

### Statistical analysis

All statistical analyses were performed using GraphPad Prism software. Unpaired two-tailed *t*-tests were performed for all experiments comparing two sets of data. For experiments with multiple comparisons ordinary one-way ANOVA was performed.

### Utilization of sequencing data

The ribo-seq data used to generate Fig. 1a was taken from Boileau et al. (*manuscript in preparation*). Ribosome profiling with Bayesian prediction version 3 (Rp-Bp v3) was used for the prediction of translated ORFs and uORF discovery (*manuscript in preparation*). Ribo-seq data used to generate Fig. 1b was taken from van Heesch et al. and analyzed using Rp-Bp^9^. The translational profiling data plotted in Supplementary Fig. 1 is taken from Boileau et al.^76^.

### Ethics statement

The iPS cell line used in this study was derived in compliance with all relevant ethical guidelines, including the principles outlined in the Declaration of Helsinki, written donor consent and institutional review board approval. All experiments were conducted in accordance with institutional and regulatory guidelines.

### Animal studies

Animal experiments were approved by the institutional animal care and use committee of Heidelberg University. All experiments were conducted in compliance with the guidelines set forth by the EU Directive 2010/63/EU and the German laws for animal protection (TierSchG and TierSchVerV).

## Electronic supplementary material

Below is the link to the electronic supplementary material.

Supplementary Material 1

Supplementary Material 2

Supplementary Material 3

## Data Availability

Sequencing data used to generate Fig. 1a is taken from Boileau et al. (manuscript in preparation). Sequencing data used to generate Fig. 1B is from van Heesch et al.^9^. The translational profiling data plotted in Supplementary Fig. 1 is from Boileau et al.^73^.
