# Supplementary material for: A novel uORF regulates folliculin to promote cell growth and lysosomal biogenesis during cardiac stress
Source: Sci Rep. 2025 Jan 27;15:3319. doi: 10.1038/s41598-025-87107-3 (PMC11770079; doi:10.1038/s41598-025-87107-3)

SUPPLEMENTARY DATA

Folliculin is regulated by a novel upstream open reading frame (uORF) to promote cell growth and lysosomal biogenesis during cardiac stress.

Maja Bencun, Laura Spreyer, Etienne Boileau, Jessica Eschenbach, Norbert Frey, Christoph Dieterich, Mirko Völkers

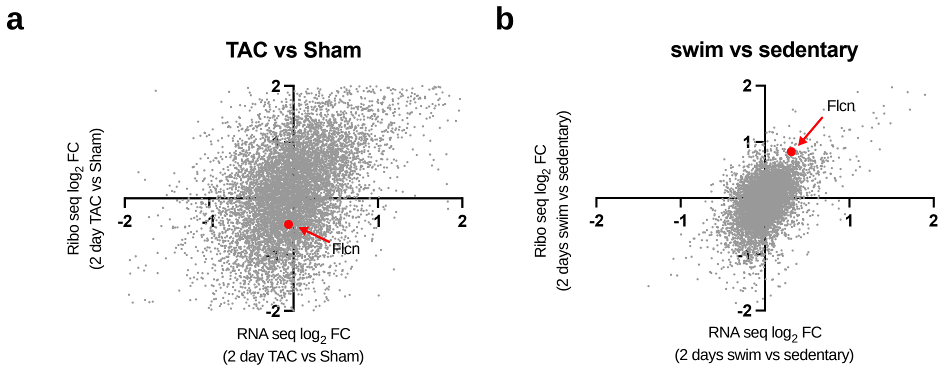

Supplementary figure 1: Flcn is post-transcriptionally regulated in the murine heart.

a. The scatter plot of mouse heart translatome (Ribo-seq) vs transcriptome (RNA-seq) data shows translational downregulation of Flcn (red dot) during pressure-overload. The plot compares the sequencing data of mice 2 days after transverse aortic constriction (TAC) surgery to 2 days after sham surgery. FC: fold change.

b. The scatter plot of mouse heart translatome (Ribo-seq) vs transcriptome (RNA-seq) data shows translational and transcriptional upregulation of Flcn (red dot) during exercise-induced hypertrophy. The plot compares the sequencing data of mice after 2 days of regular swimming exercise to 2 days of sedentary behavior. FC: fold change.

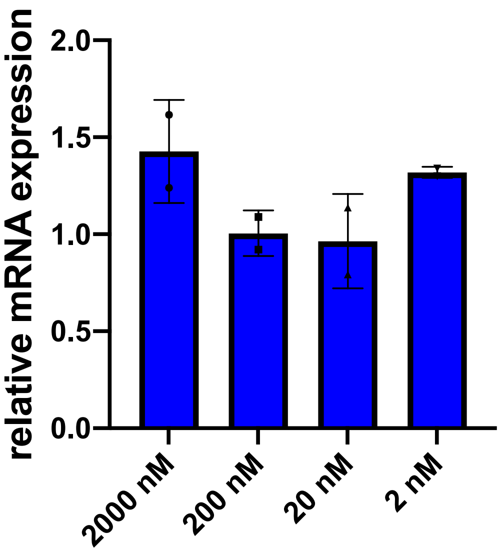

Supplementary figure 2: FLCN mRNA expression levels following ASO transfection.

The bar graph depicts FLCN mRNA levels following transfection of HeLa cells with an antisense oligonucleotide (ASO) targeting the uORF encoded on the 5'UTR of FLCN mRNA. *n = 2 replicates*

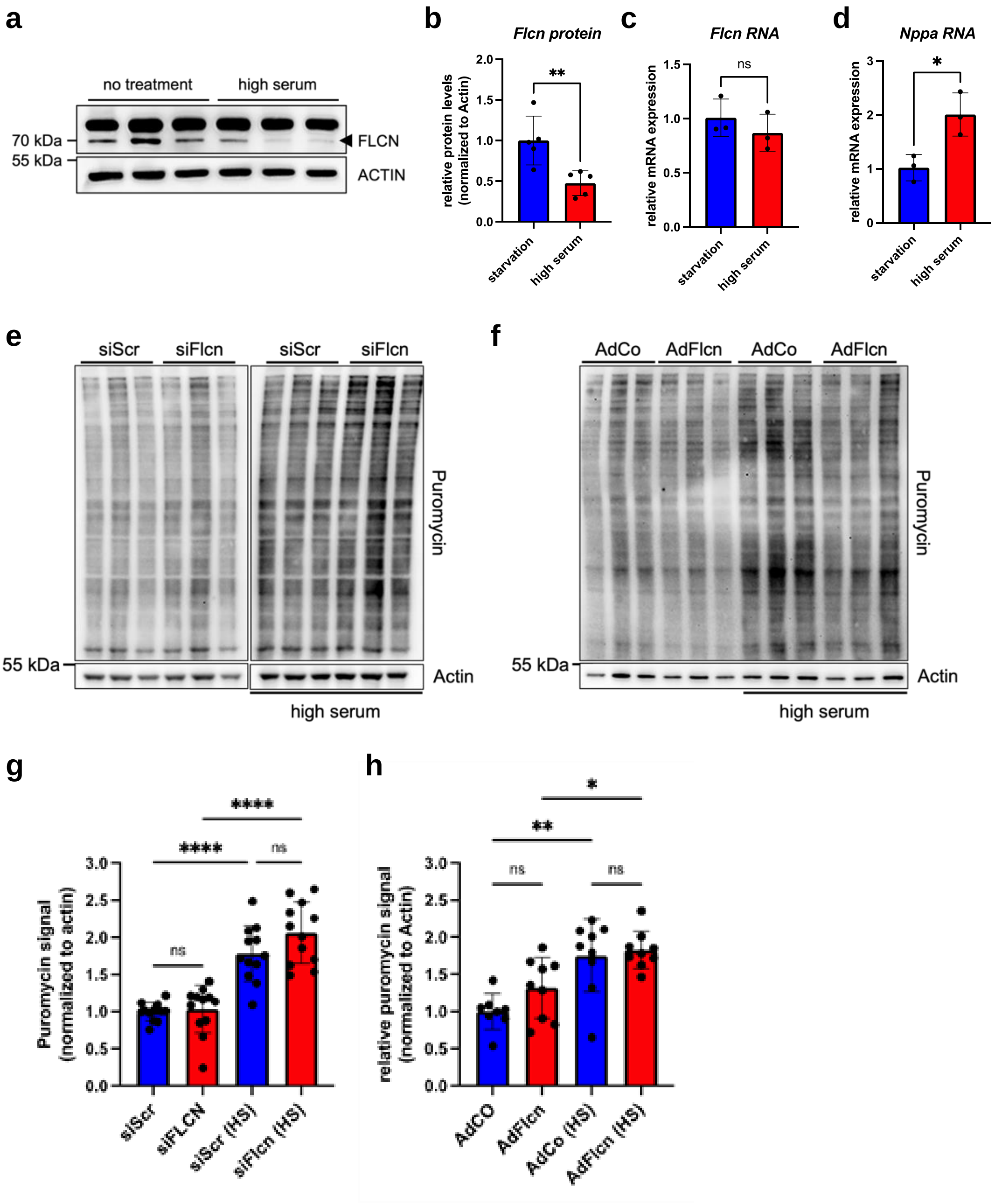

Supplementary figure 3: Flcn protein levels are downregulated by high serum stimulation. a. Flcn protein levels in neonatal rat cardiomyocytes (NRCMs) before and after high serum stimulation are shown. Actin was used as loading control. b. The bar graph shows the quantification of band intensities from a. Band intensities were normalized to Actin levels. c-d. The bar graph shows the relative mRNA levels of Flcn and Nppa in NRCMs following high serum stimulation. e. Representative immunoblots of the puromycin incorporation assay in NRCMs after transfection of scrambled (siScr) or Flcn-targeting (siFlcn) siRNA followed by stimulation with high serum for 24h. f. Representative immunoblots of the puromycin incorporation assay of NRCMs after adenoviral transduction with control Adenovirus (AdCo) or Flcn-overexpressing virus (AdFlcn) followed by stimulation with high serum for 24h. g+h. Quantification of the immunoblots shown in e+f. Puromycin incorporation was normalized to actin band intensities.

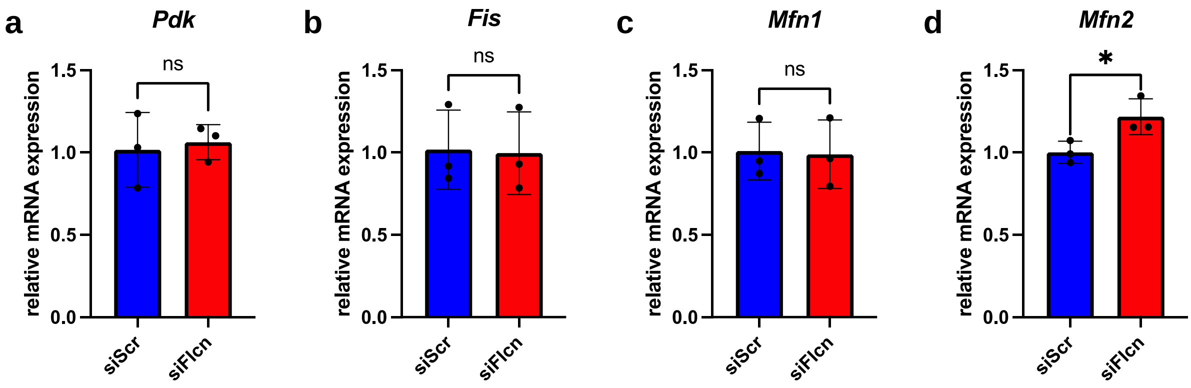

Supplementary figure 4: Mitochondrial marker gene expression following Flcn knockdown in neonatal rat cardiomyocytes (NRCMs).
The bar graphs show the relative mRNA levels of mitochondrial marker genes in NRCMs with and without Flcn KD: a. pyruvate dehydrogenase (Pdk) b. mitochondrial fission 1 protein (Fis1) c. mitofusin 1 (Mfn1) d. mitofusin 2 (Mfn2). siScr: scrambled siRNA control. siFlcn: Flcn-targeting siRNA. Data was analyzed by unpaired t-test. Error bars indicate ± standard deviation. ns: not significant. **p*-value ≤ 0.01. *n= 3 replicates*

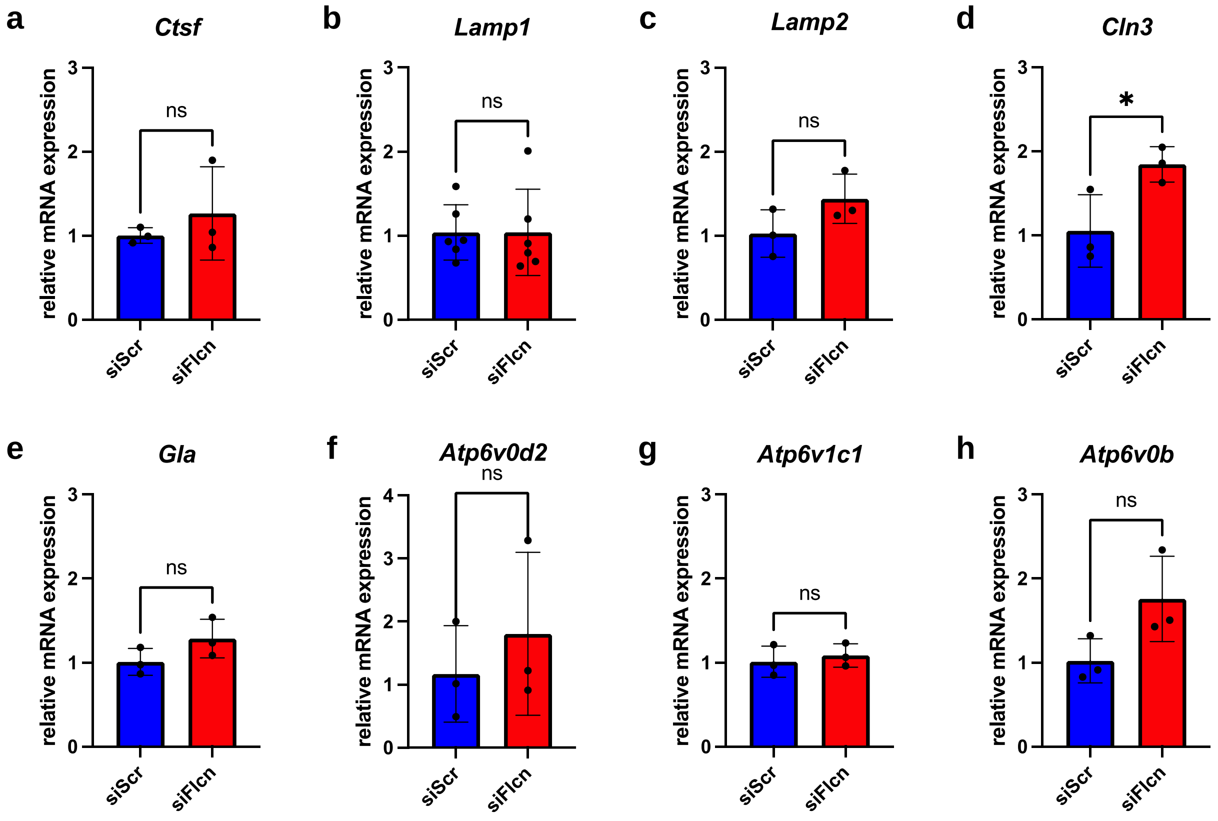

Supplementary figure 5: Quantification of lysosomal marker gene expression by RT-qPCR following Flcn knockdown in NRCMs.

The bar graphs show the relative mRNA levels of lysosomal marker genes in NRCMs with and without Flcn KD. a. Cathepsin F (Ctsf) b. Lysosomal associated membrane protein 1 (Lamp1) c. Lysosomal associated membrane protein 2 (Lamp2) d. Cln3 lysosomal/endosomal transmembrane protein, battenin (Cln3) e. Galactosidase alpha (Gla) f. (Atp6v0d2) g. V-type protein ATPase subunit C1 (Atp6v1c1) h. ATPase H+ transporting V0 subunit b (Atp6v0b). siScr: scrambled siRNA control. siFlcn: Flcn-targeting siRNA. Data was analyzed by unpaired t-test. Error bars indicate ± standard deviation. ns: not significant. *p-value ≤ 0.01. *n= 3 replicates*

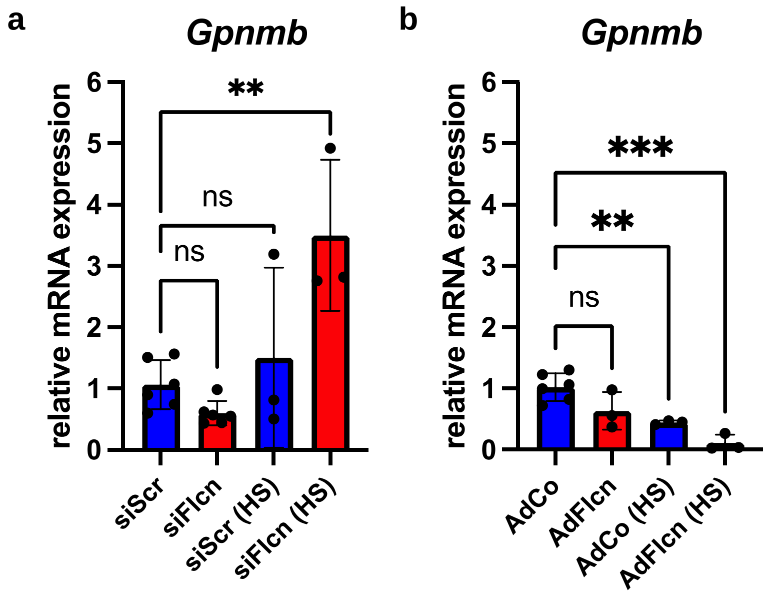

Supplementary figure 6: Quantification of lysosomal marker gene expression by RT-qPCR following Flcn knockdown knockdown in neonatal rat cardiomoyocytes (NRCMs).

The bar graphs show the relative mRNA levels of the glycoprpotein nonmetastatic melanoma B gene (Gpnmb) in NRCMs following Flcn KD (a) and overexpression (b) with and without high serum stimulation (HS). siScr: scrambled siRNA control. siFlcn: Flcn-targeting siRNA. AdCo: control adenovirus (empty overexpression vector). AdFlcn: Flcn overexpressing adenovirus. Data was analyzed by one-way ANOVA. Error bars indicate ± standard deviation. ns: not significant. **p*-value ≤ 0.01. ***p*-value ≤ 0.001. *n= 3 replicates*

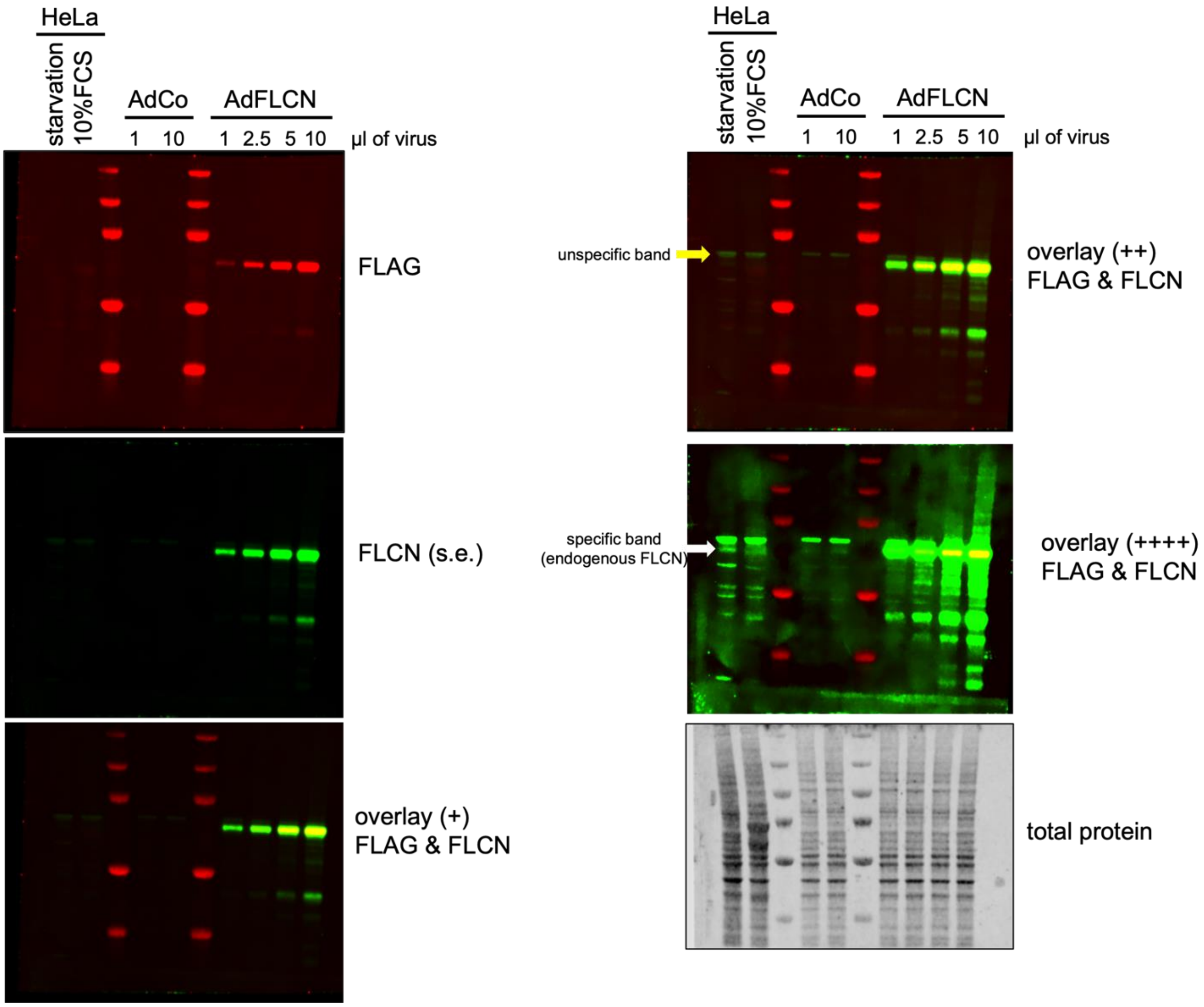

Supplementary figure 7: Validation of FLCN antibody for Western blot.

Hela cells were transduced with 3xFLAG-tagged FLCN-encoding or control adenovirus. HeLa cells were cultured without FCS (starvation) for 4 hours to upregulate endogenous FLCN in the control lysates. Blot was probed with FLAG tag antibody (red) and FLCN (green) to help identify the specific band.

Uncropped blots – related to Figure 2e

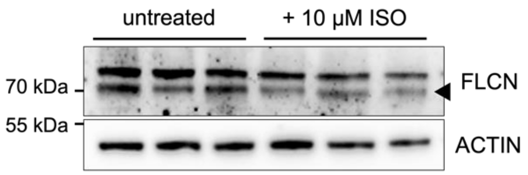

Exposures used for the image:

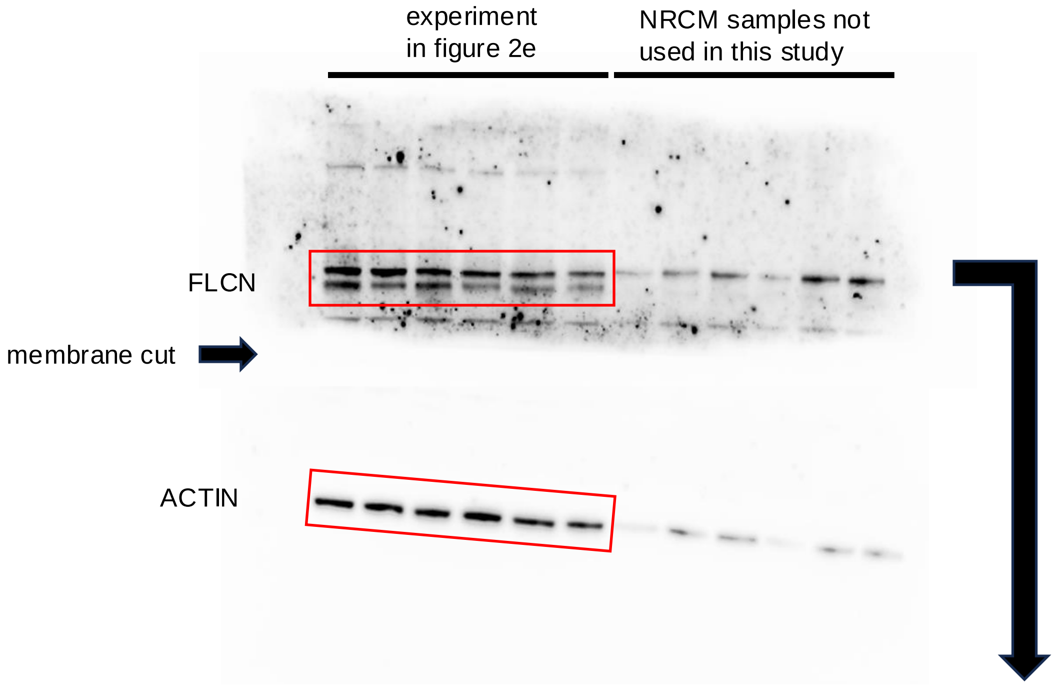

Membrane overview:

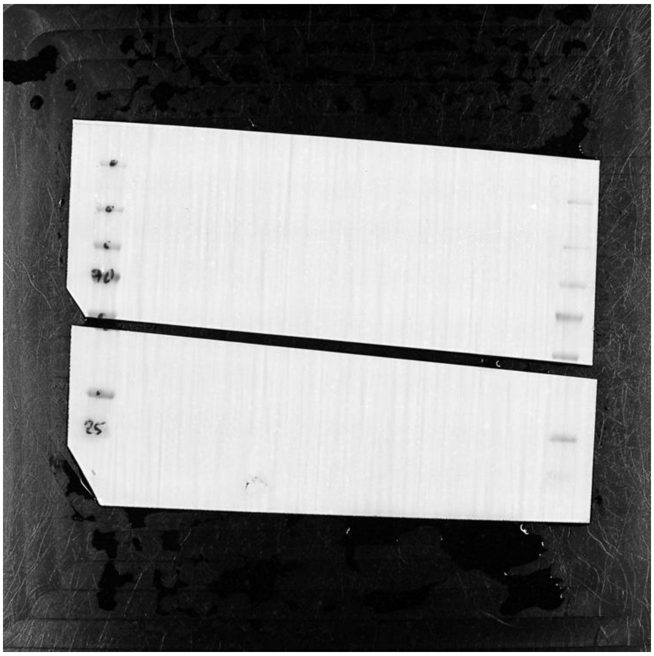

Membrane image merged FLCN exposure image:

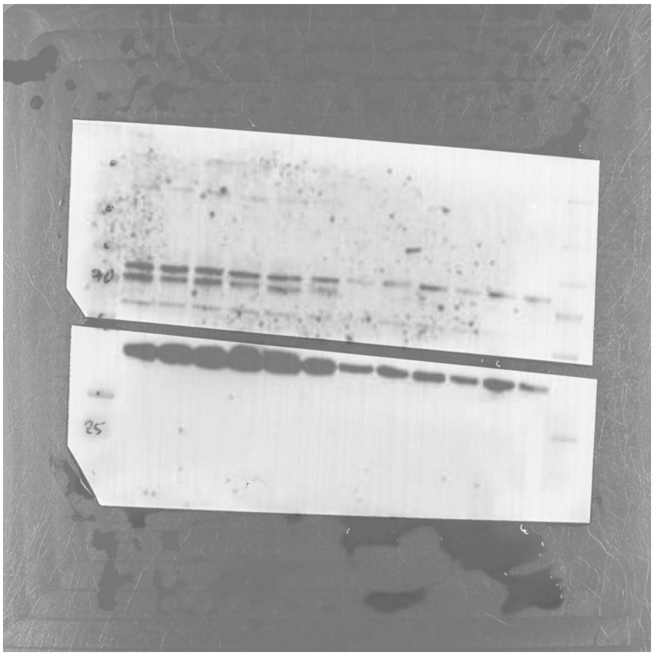

Uncropped blots – related to Figure 2e

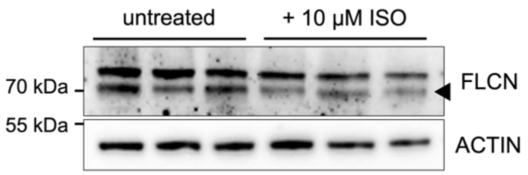

Exposures used for the image:

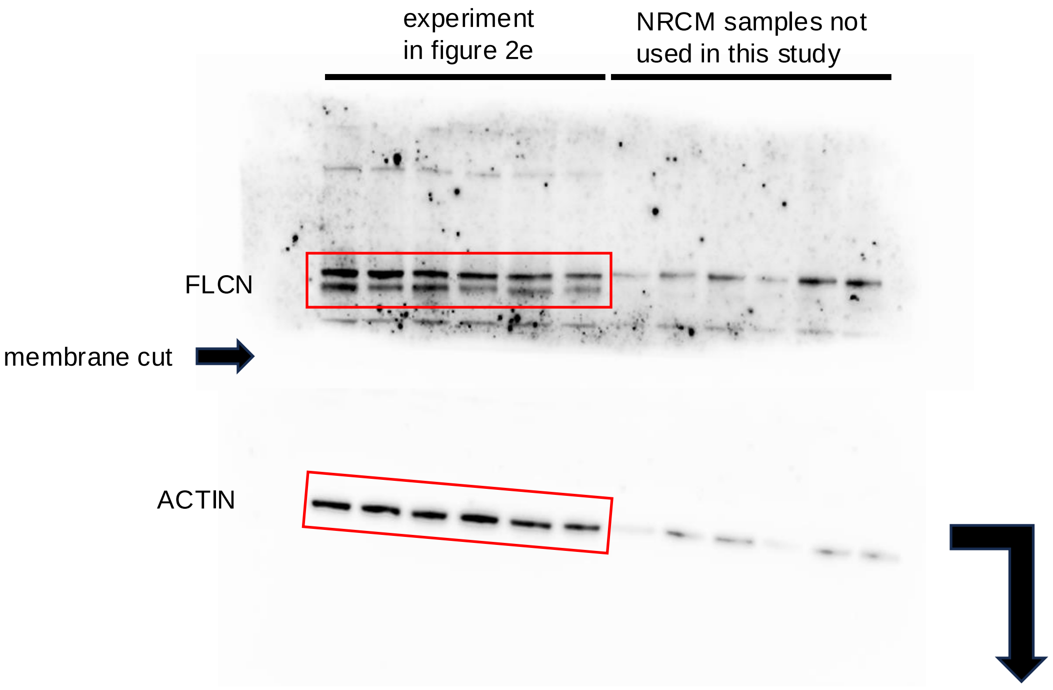

Membrane overview:

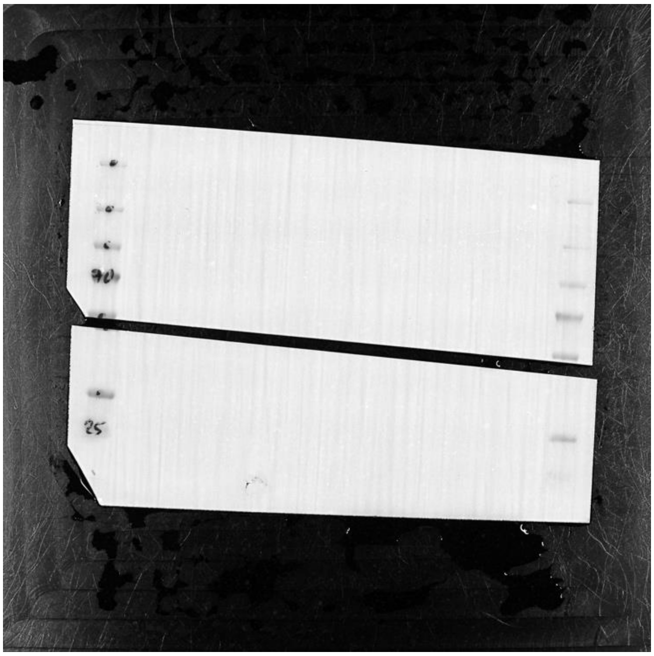

Membrane image merged with ACTIN exposure image:

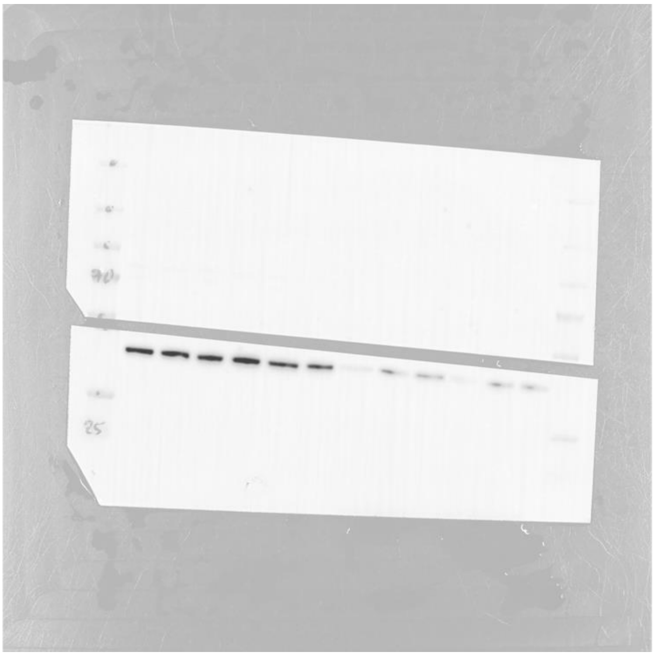

Uncropped blots – related to Figure 2h

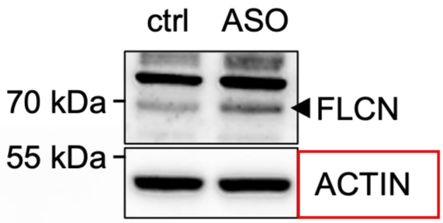

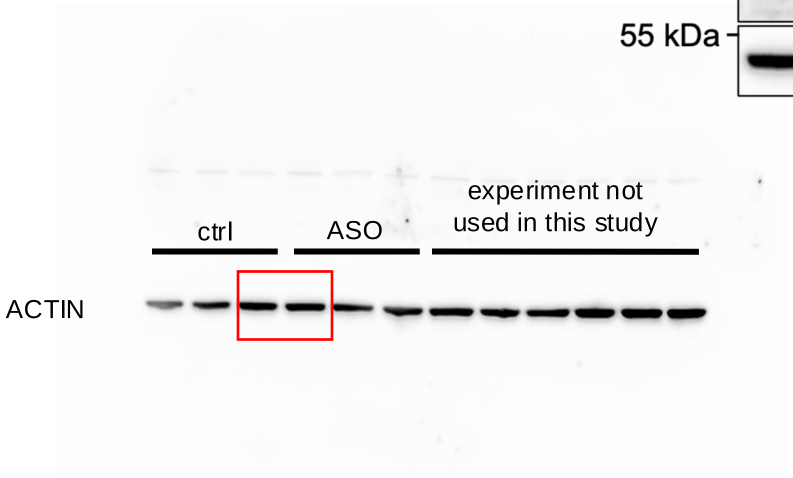

Membrane overview:

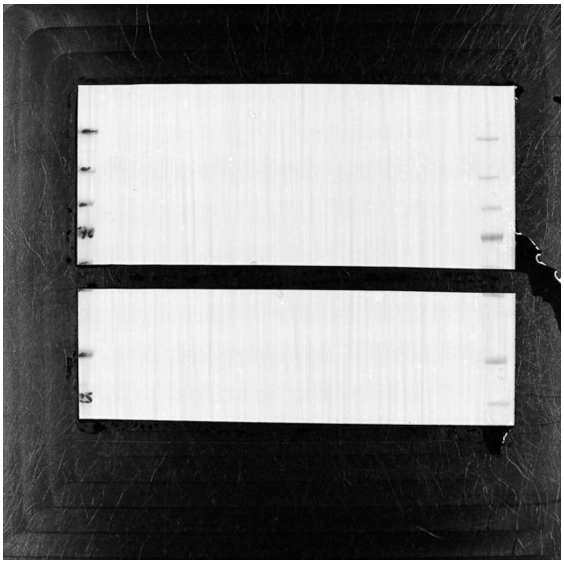

Membrane image merged with ACTIN exposure image:

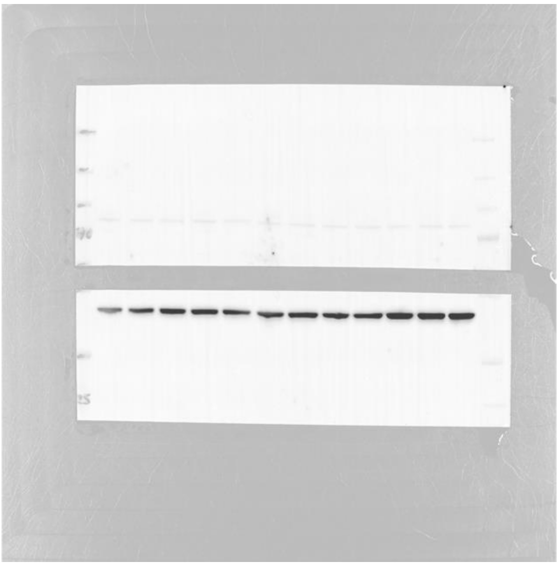

Strongest exposure time:

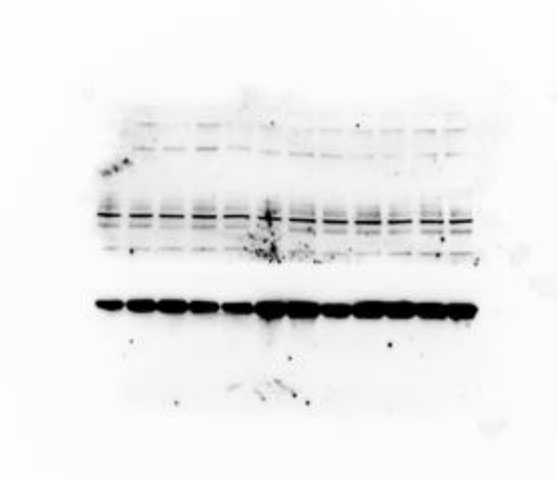

Membrane image merged with strongest exposure image (shown on the left):

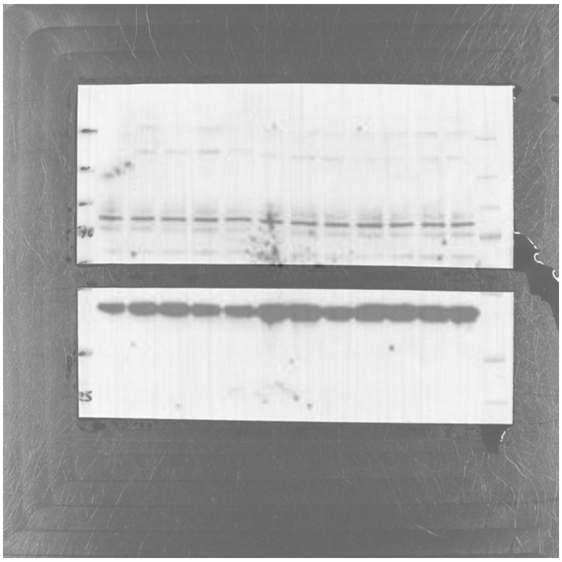

The 6th lane exhibits background signal making quantification of the FLCN band difficult → the upper part of the membrane was washed and redeveloped to remove background (see next page)

Uncropped blots – related to Figure 2h

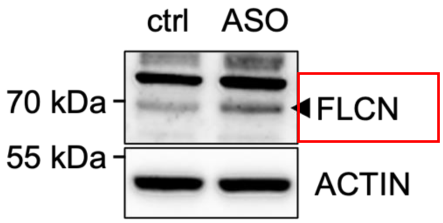

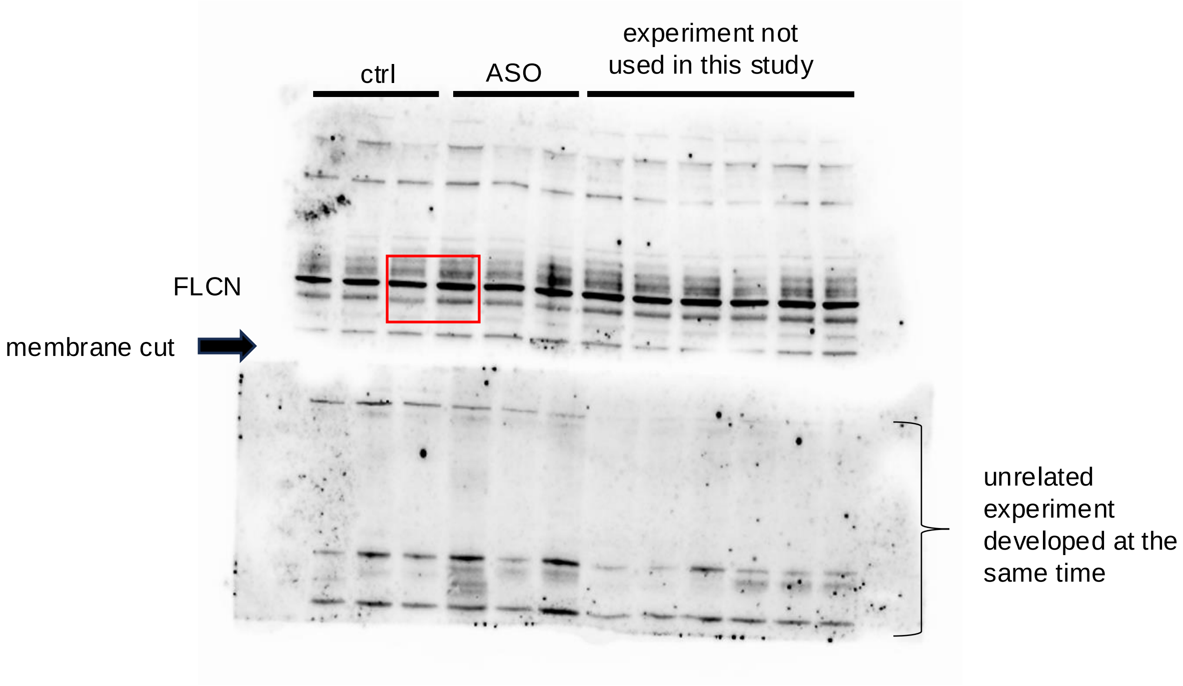

Membrane overview:

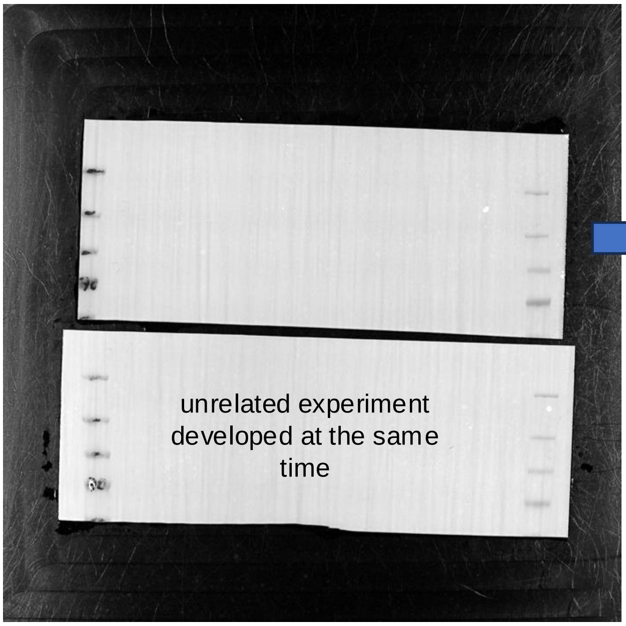

Membrane image merged with FLCN exposure image:

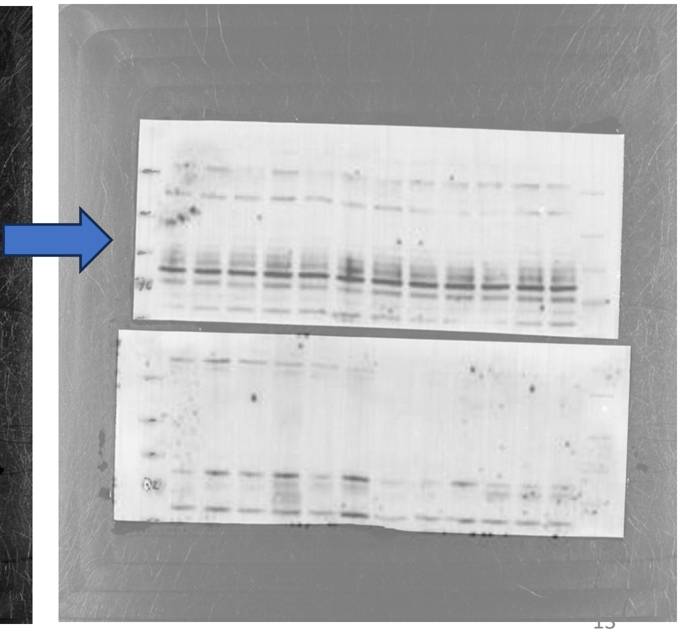

Uncropped blots – related to Figure 3a

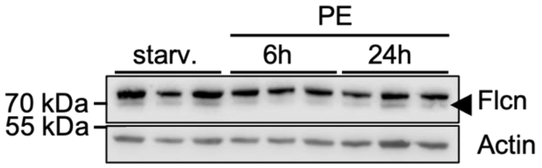

<u>long exposure time:</u>
(used to show Flcn protein levels)

Flcn

membrane cut

Actin

membrane cut

p-4E-BP1
(not used in manuscript)

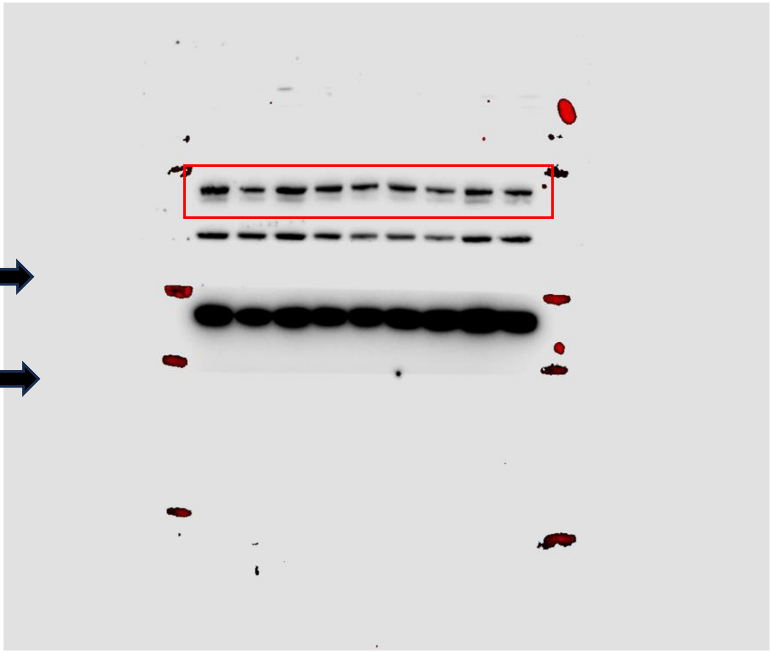

<u>shorter exposure time:</u>
(used to show Actin protein levels)

Flcn

membrane cut

Actin

membrane cut

p-4E-BP1
(not used in manuscript)

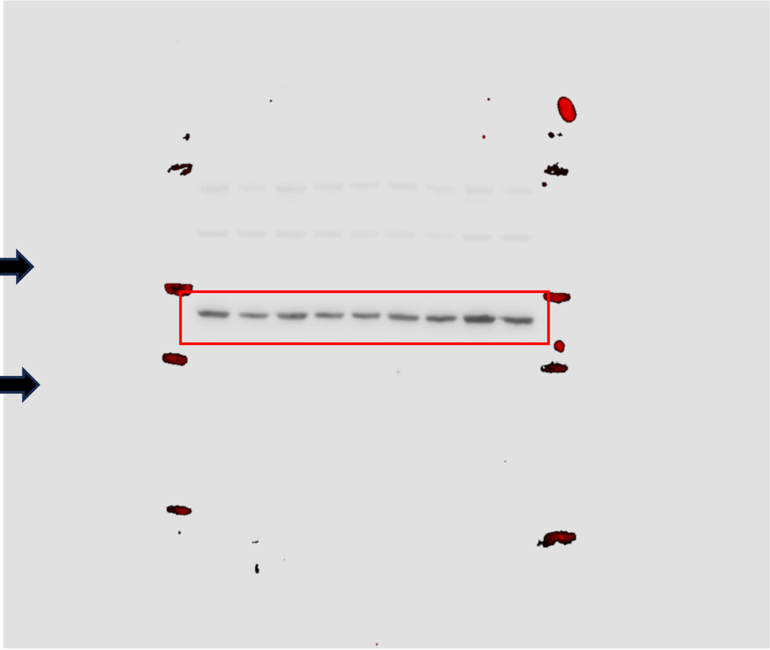

Uncropped blots – related to Figure 3a

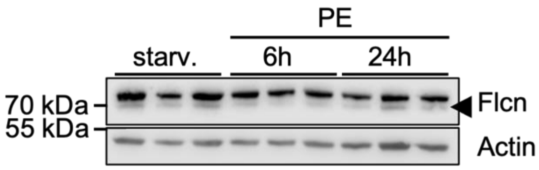

<u>overexposed:</u>
(to show membrane borders)

Flcn

membrane cut

Actin

membrane cut

p-4E-BP1
(not used in manuscript)

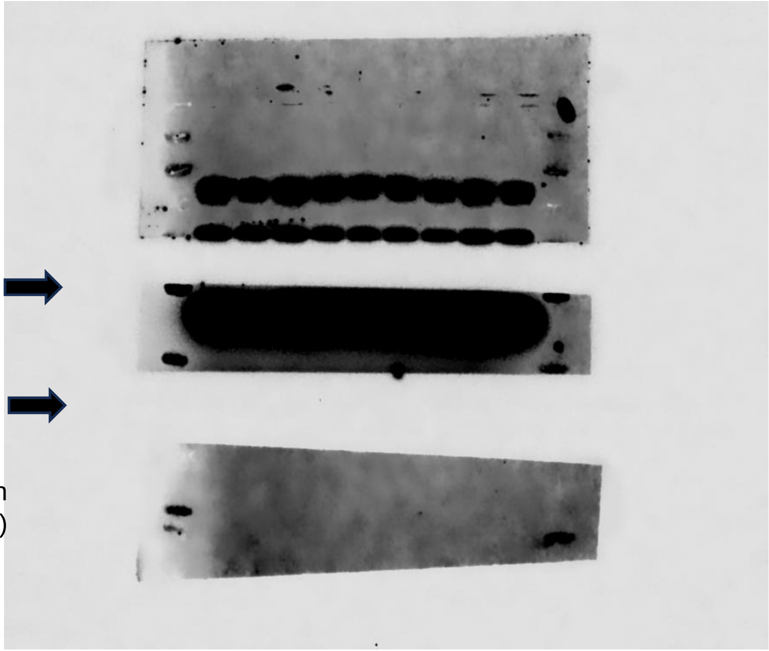

Uncropped blots – related to Figure 3h

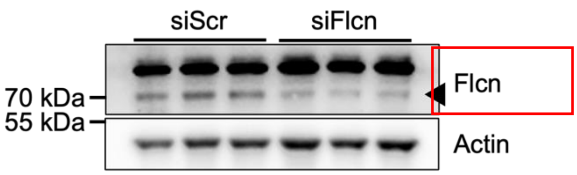

Overexpression of Flcn with different stimuli (not used – overexpression did not work)

Flcn

membrane cut

siRNA KD of Flcn with different stimuli

Flcn

membrane cut

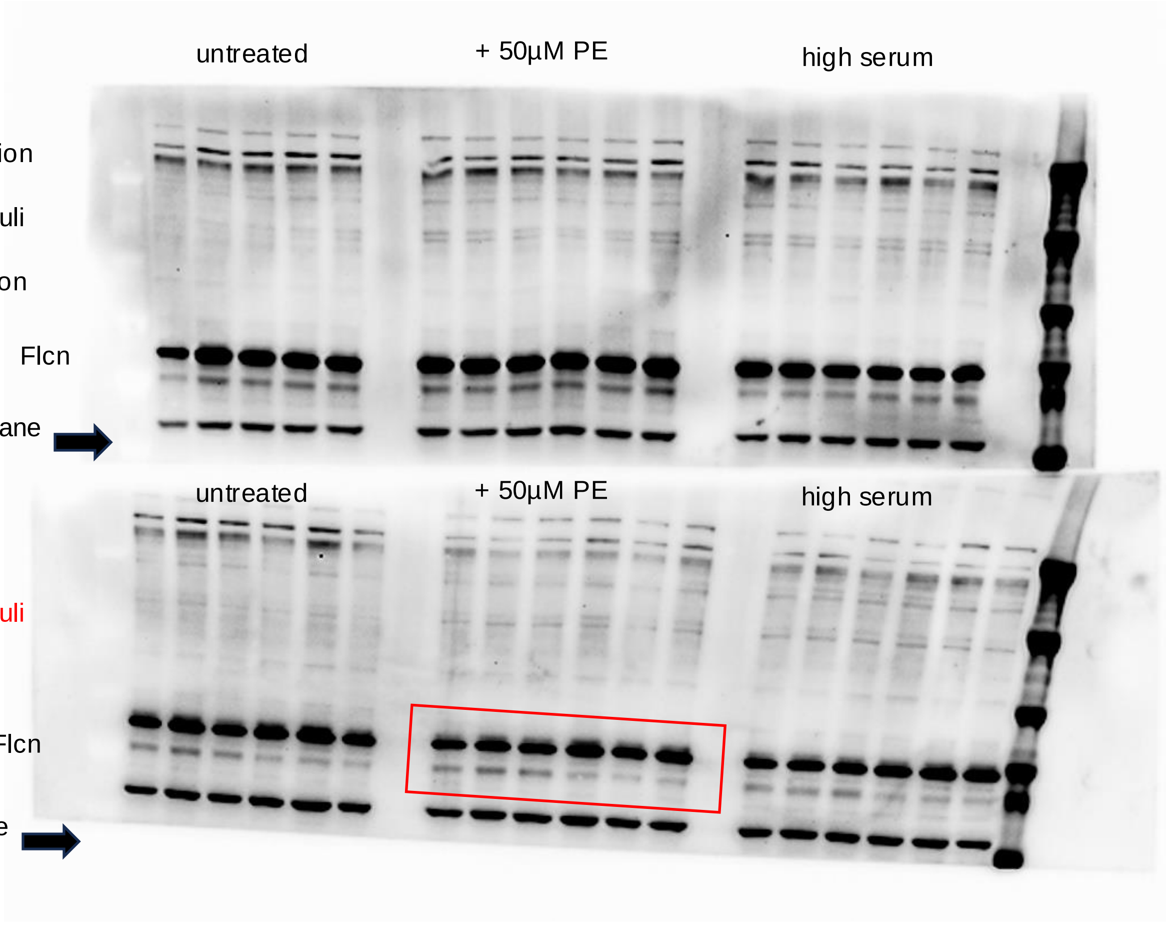

Membrane overview:

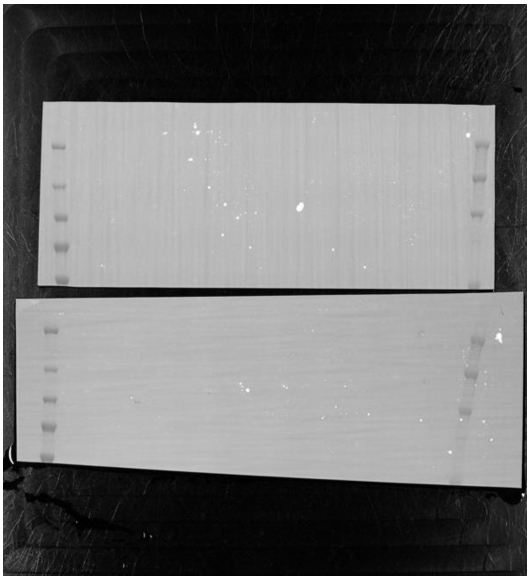

Membrane image merged with Flcn exposure image:

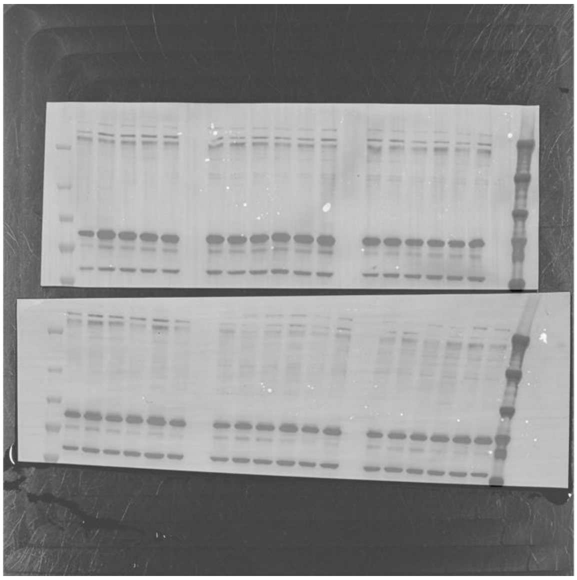

Uncropped blots – related to Figure 3h

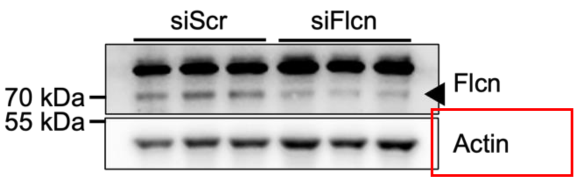

Overexpression of Flcn with different stimuli (not shown in figures)

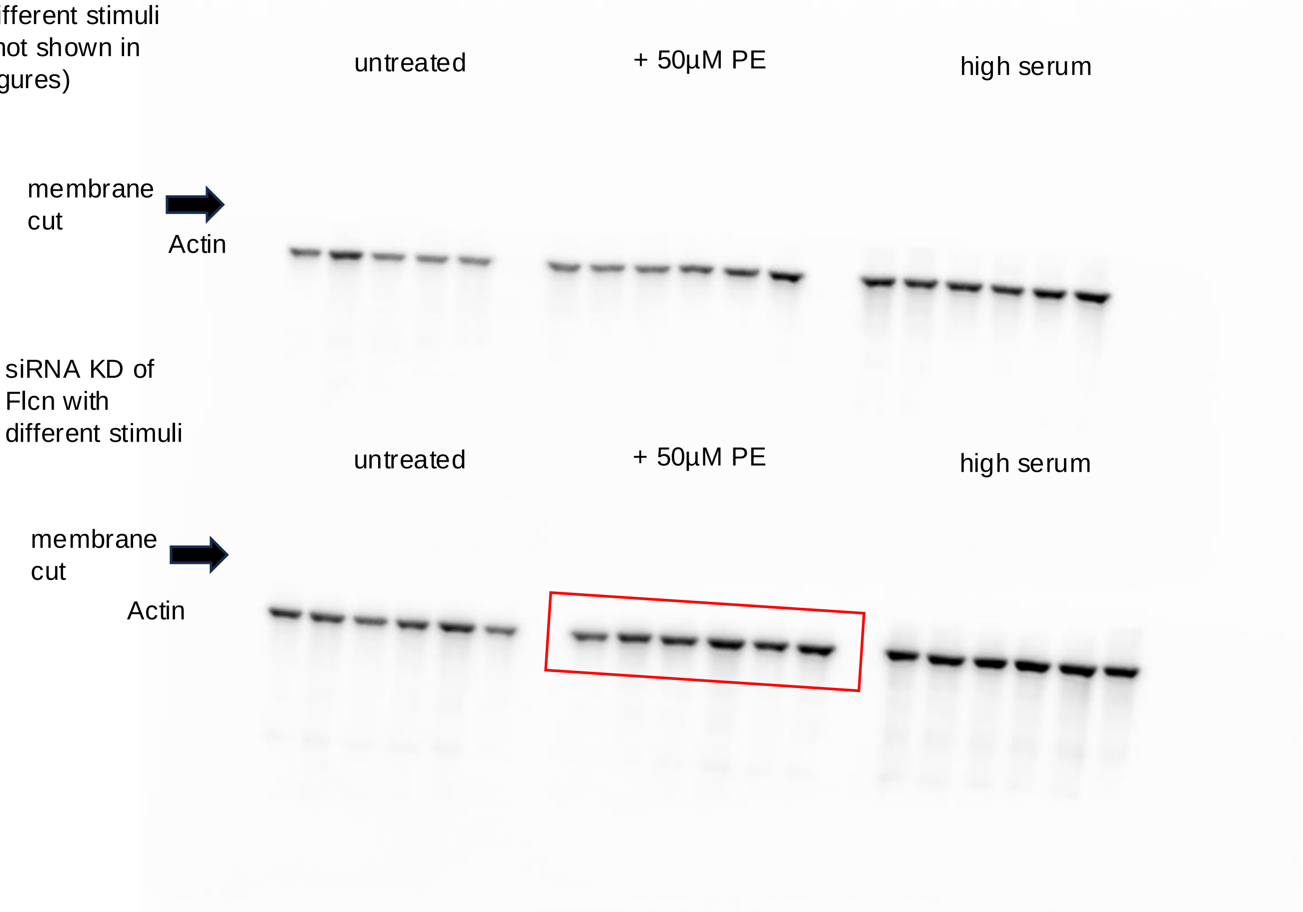

Membrane overview:

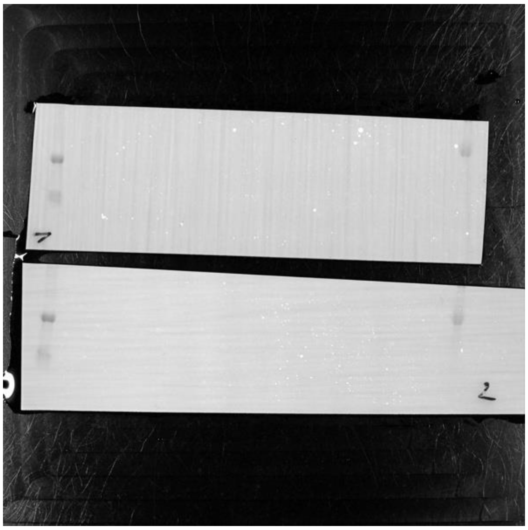

Membrane image merged with Actin exposure image:

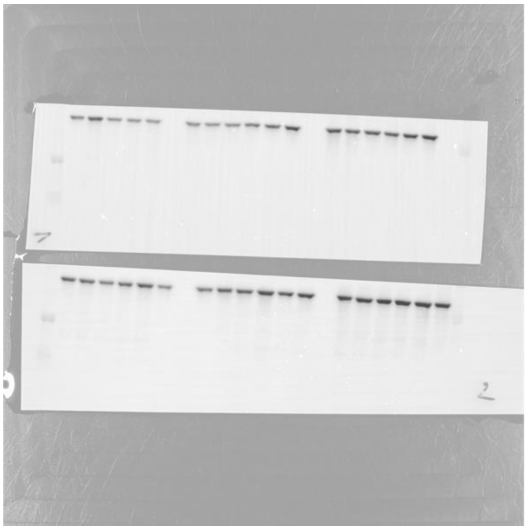

Uncropped blots – related to Figure 3n

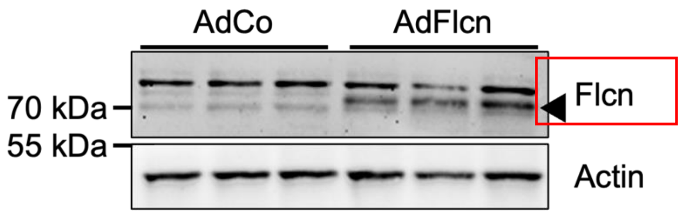

Overexpression of Flcn with different stimuli

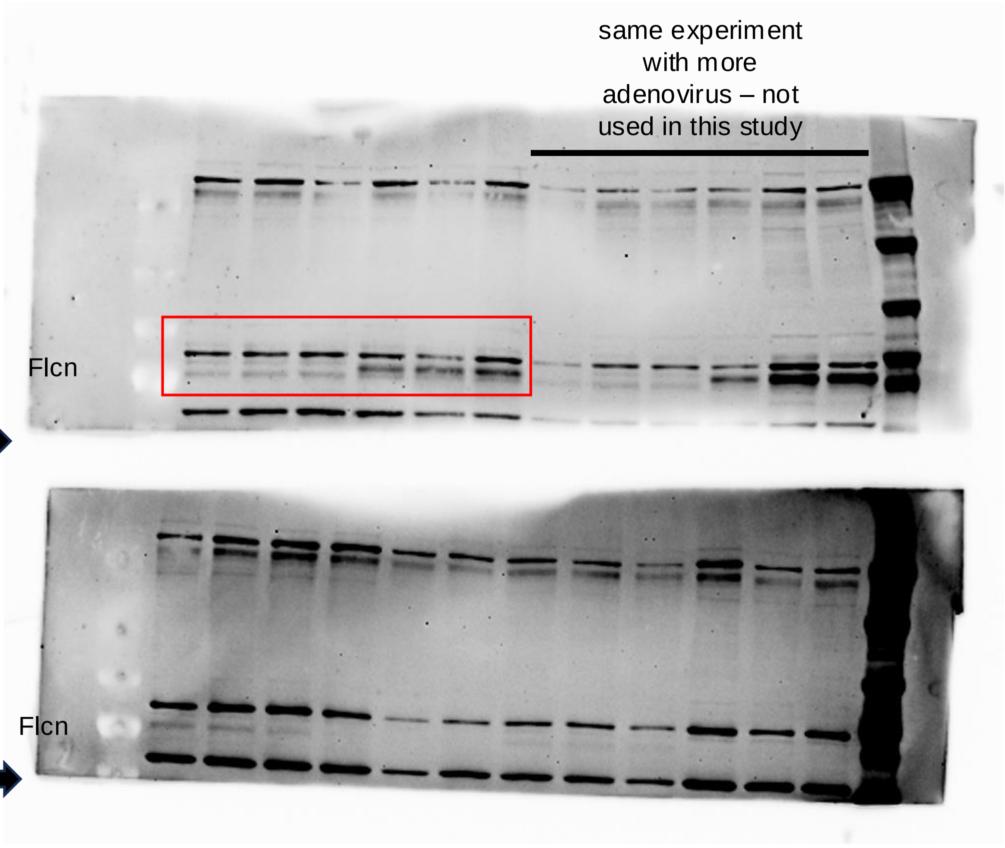

membrane cut

siRNA KD of Flcn with (notshown in figures)

membrane cut

Membrane overview:

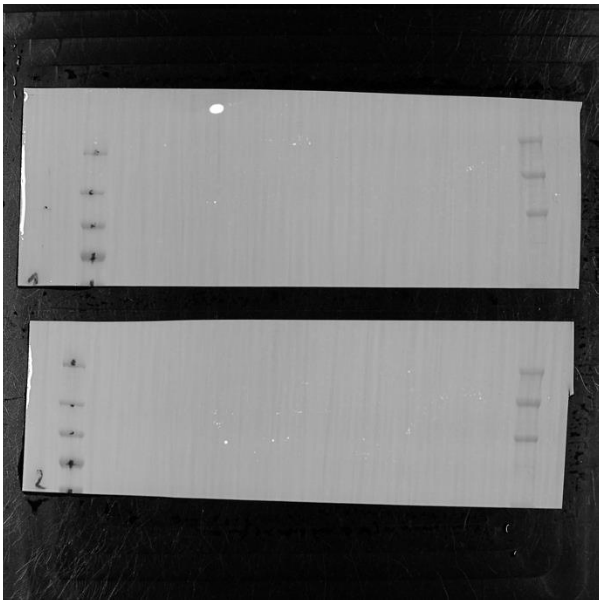

Membrane image merged with Flcn exposure image:

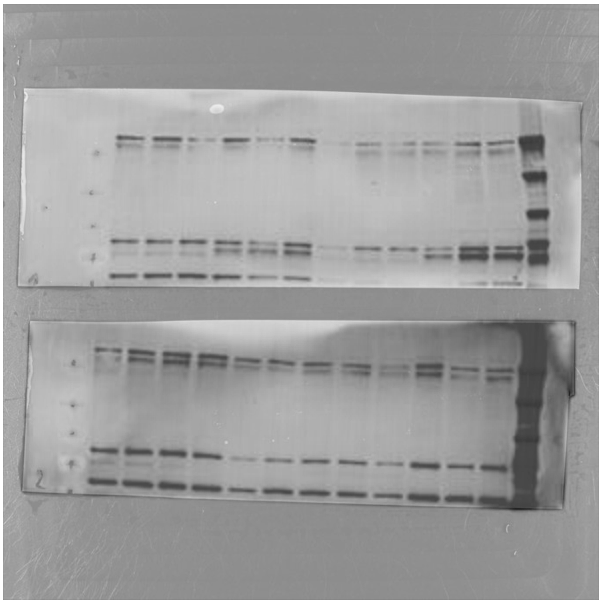

Uncropped blots – related to Figure 3n

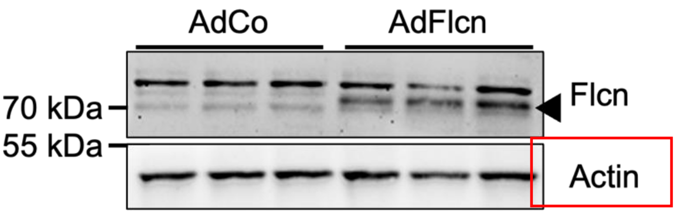

Overexpression of Flcn with different stimuli

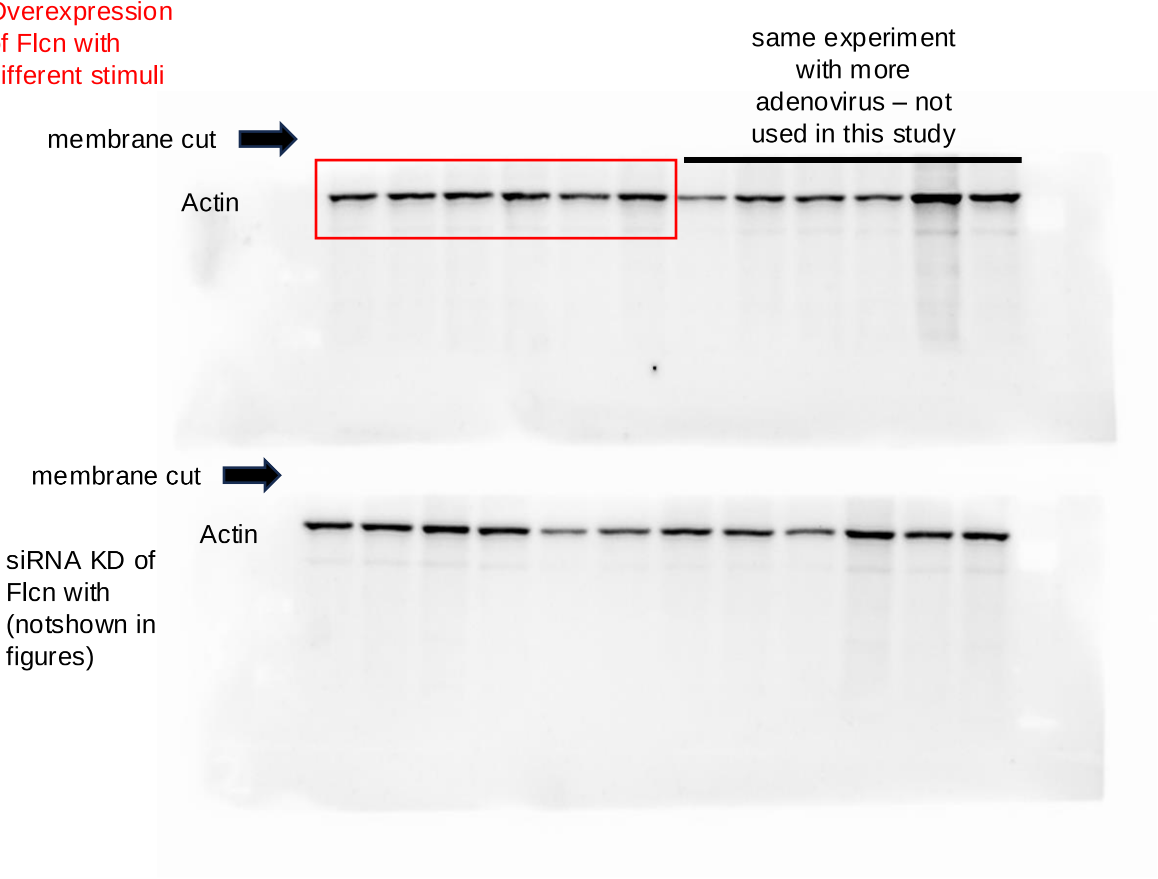

Membrane overview:

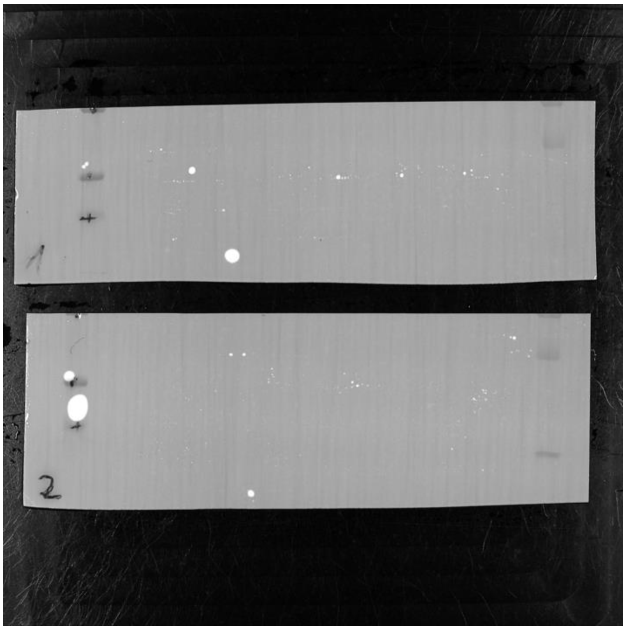

Membrane image merged with Actin exposure image:

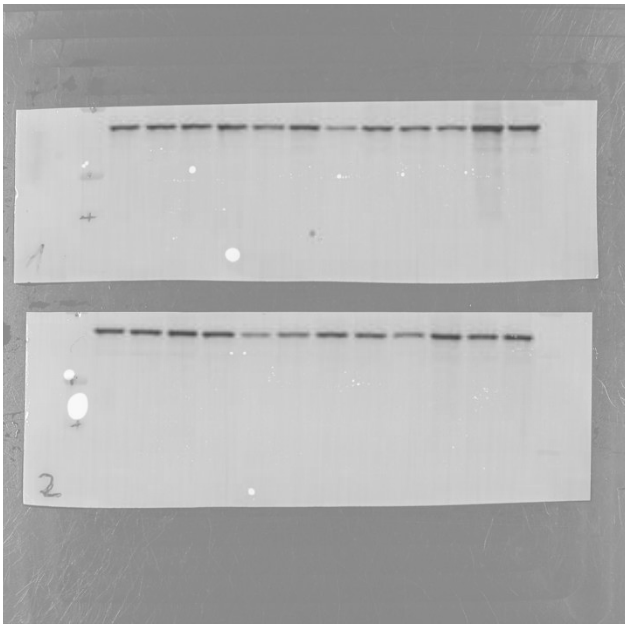

Uncropped blots – related to Figure 4b

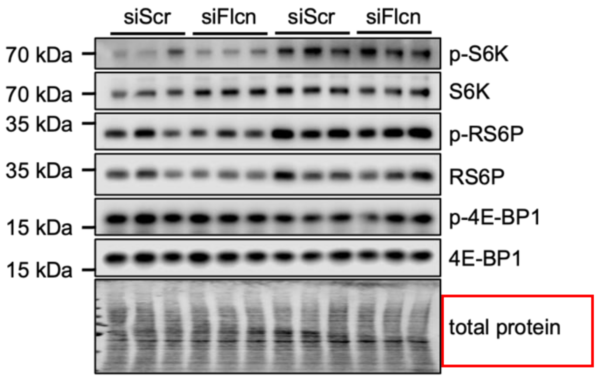

total protein stain

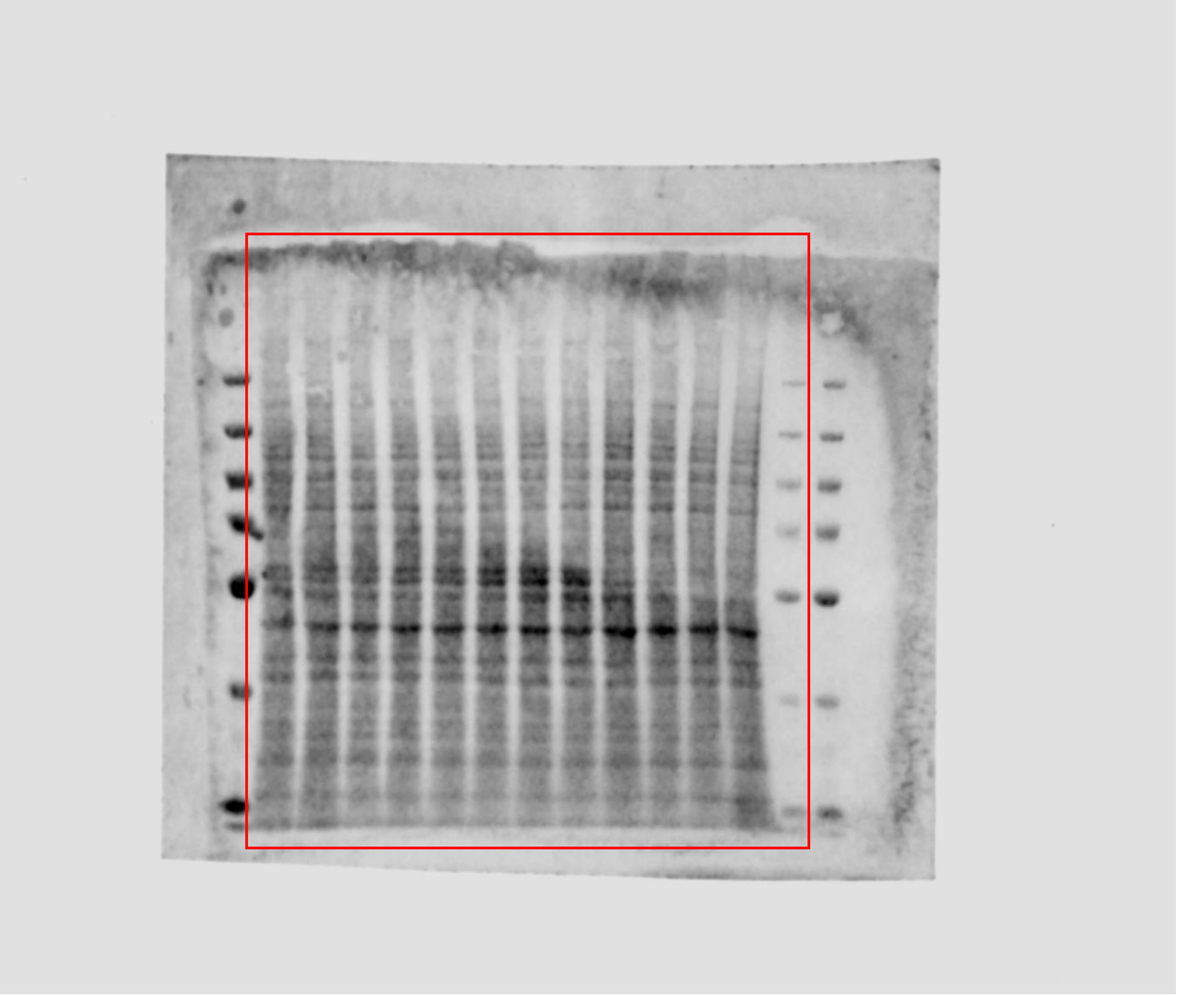

Uncropped blots – related to Figure 4b

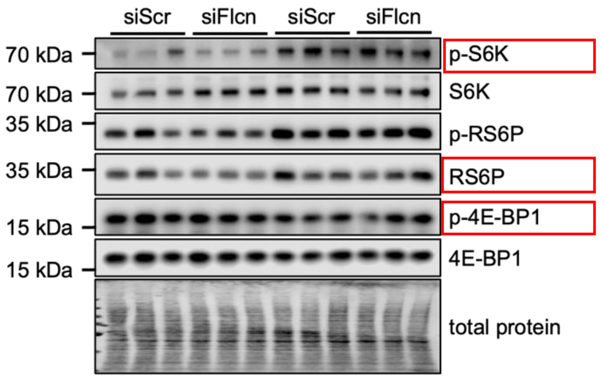

Strong exposure with infrared markers visible (red):

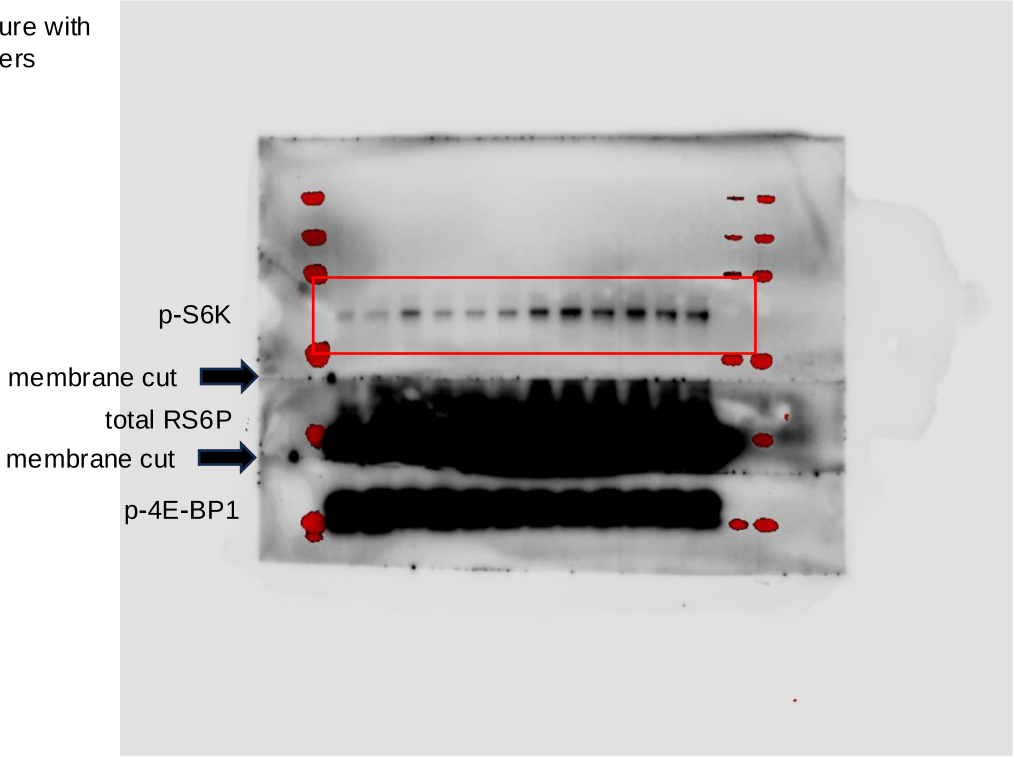

shorter exposure times:

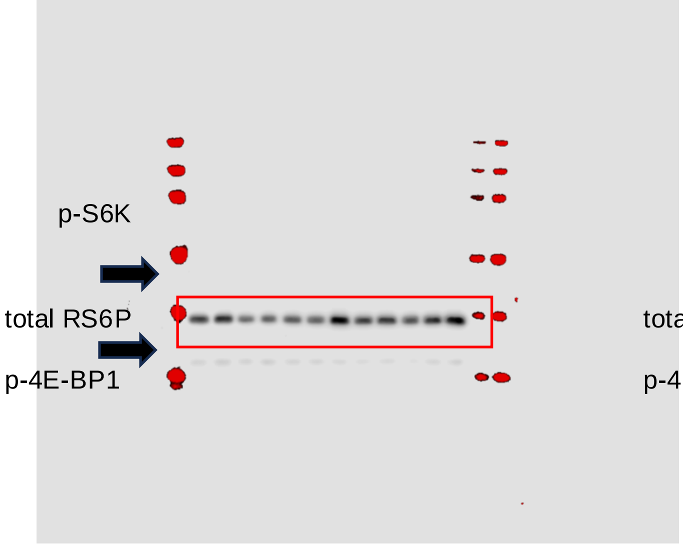

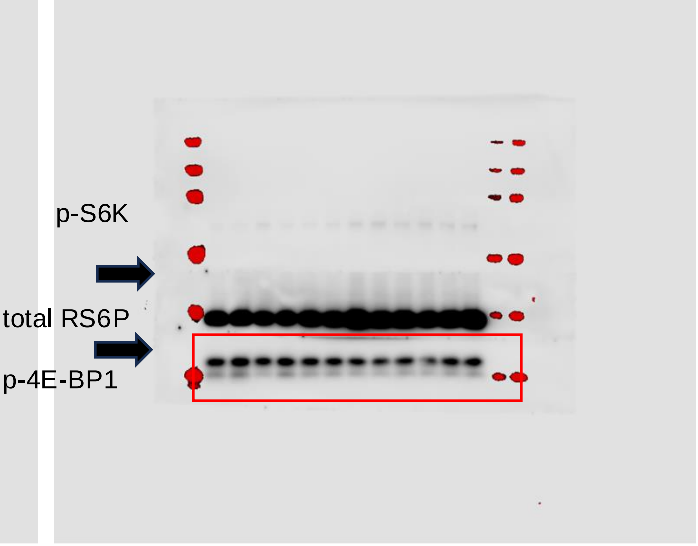

Uncropped blots – related to Figure 4b

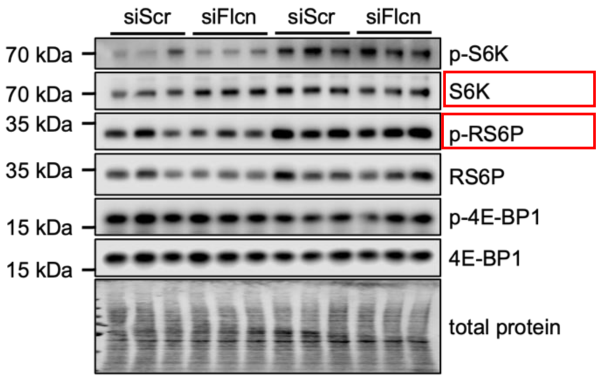

Strong exposure with infrared markers visible (red):

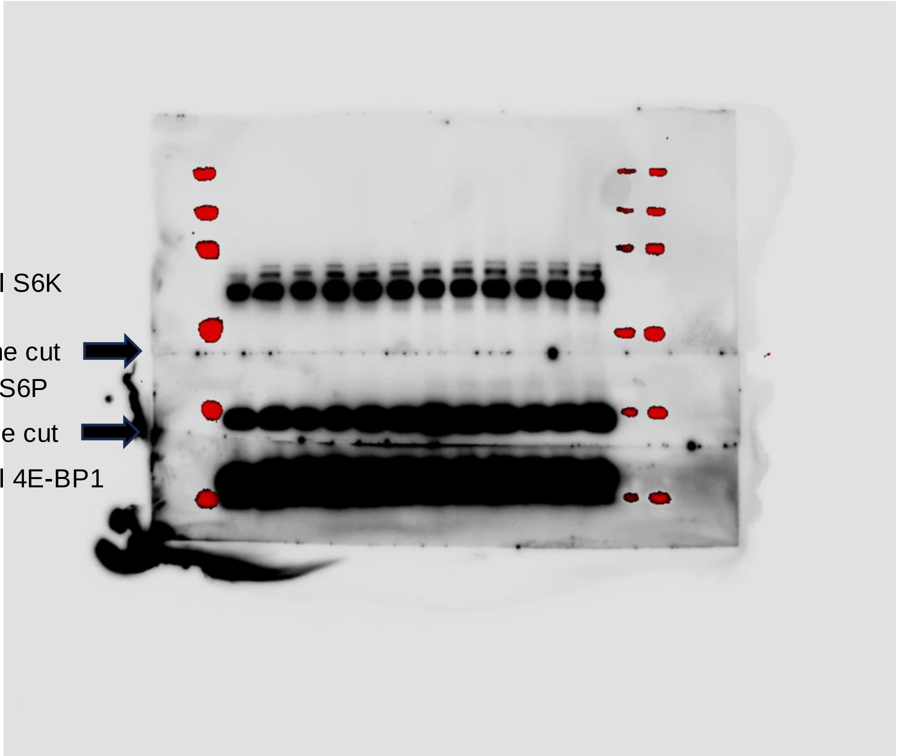

shorter exposure times:

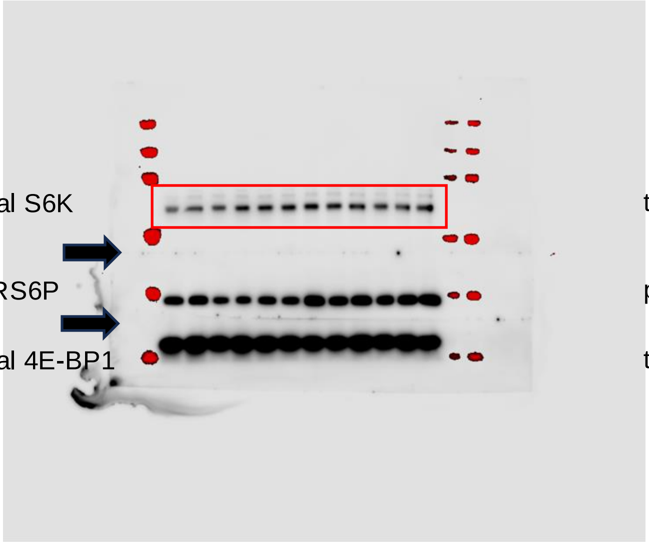

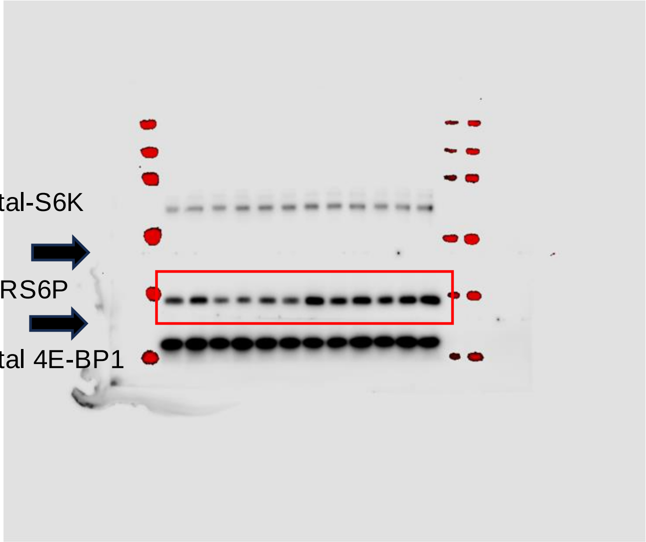

Uncropped blots – related to Figure 4b

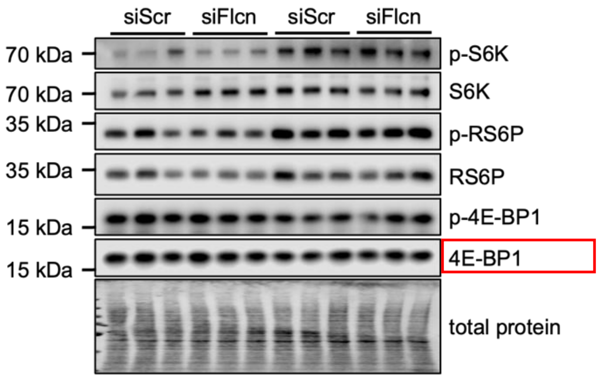

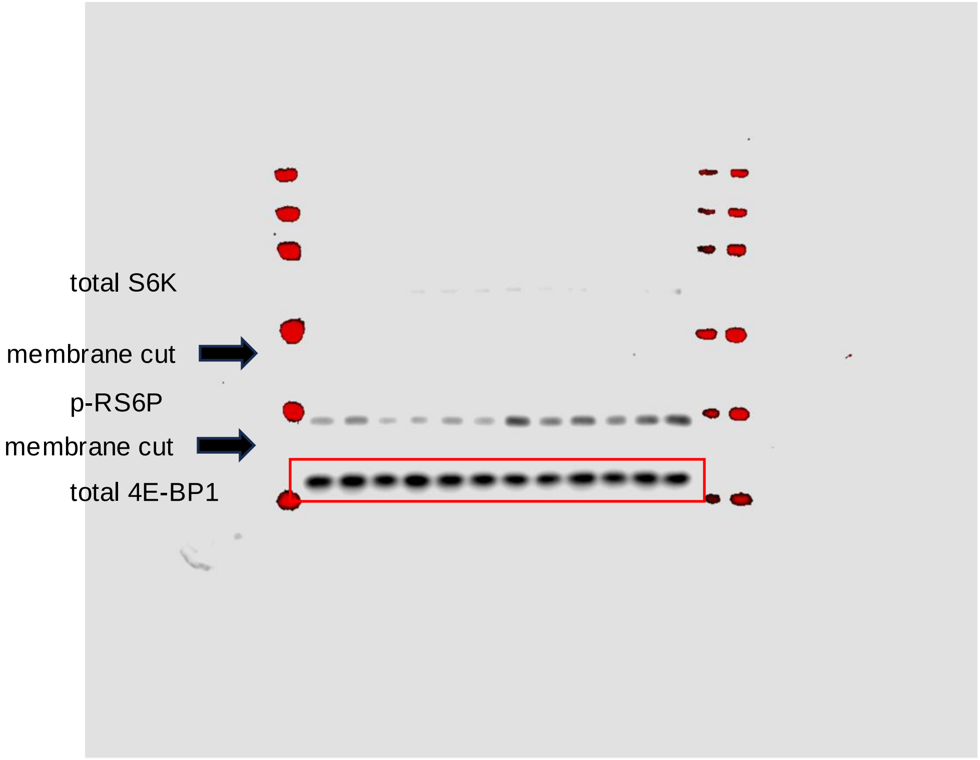

Uncropped blots – related to Figure 4f

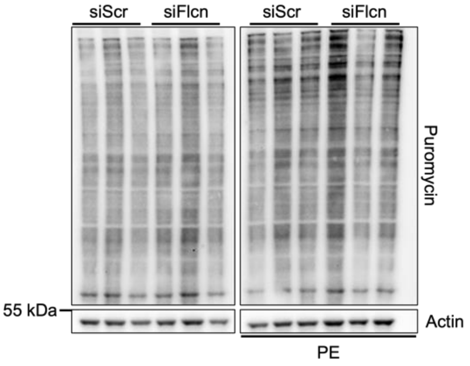

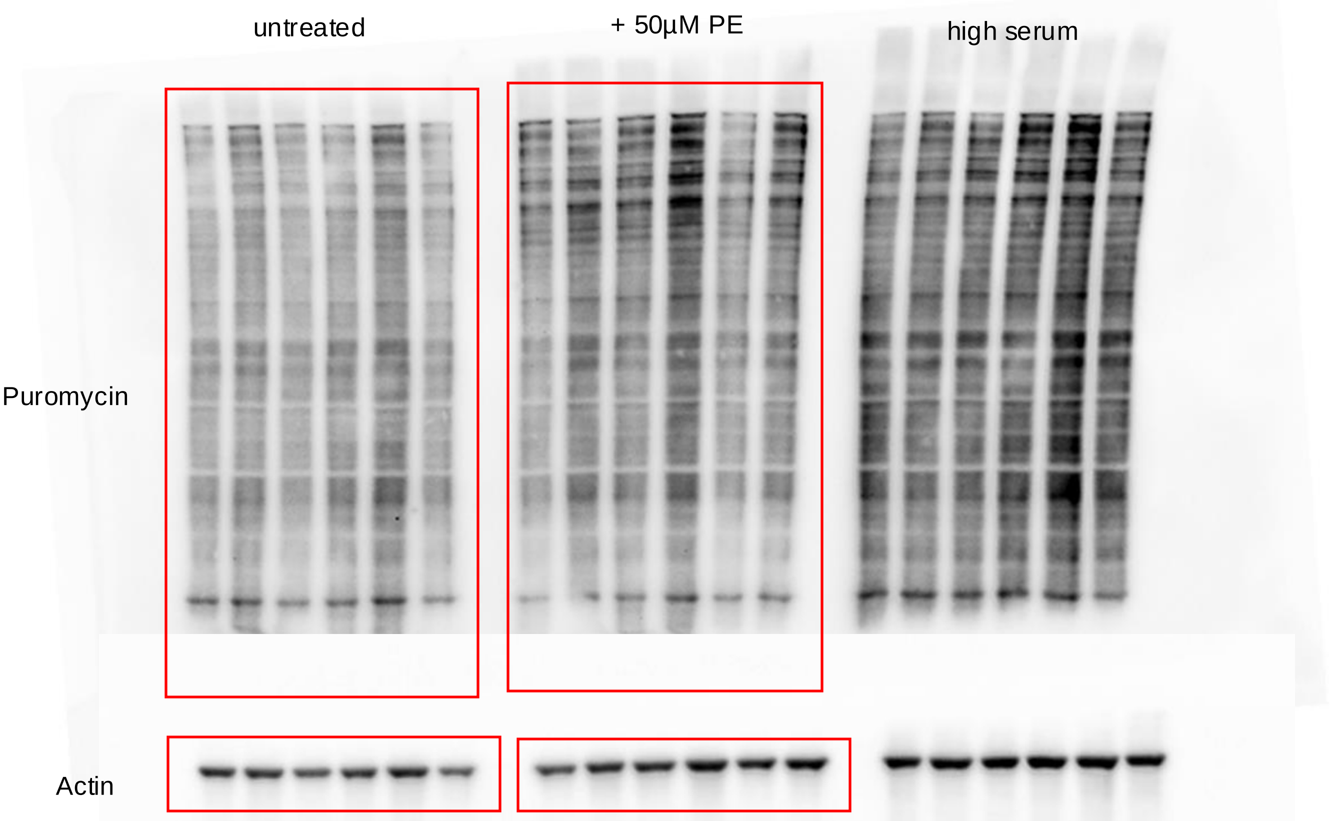

for membrane overlays of Actin blots see supplementary data page 18

Membrane overview:

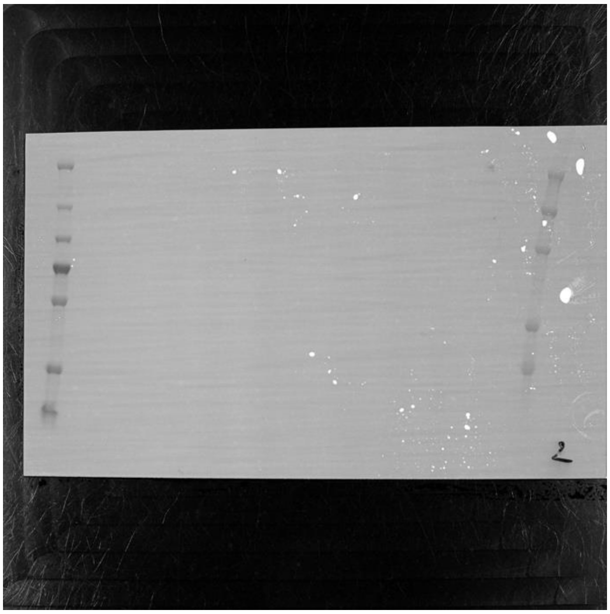

Membrane image merged with puromycin exposure image:

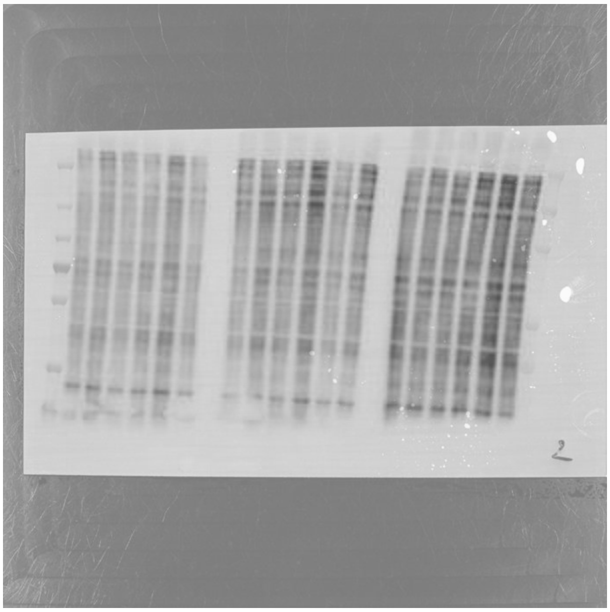

Uncropped blots – related to Figure 4h

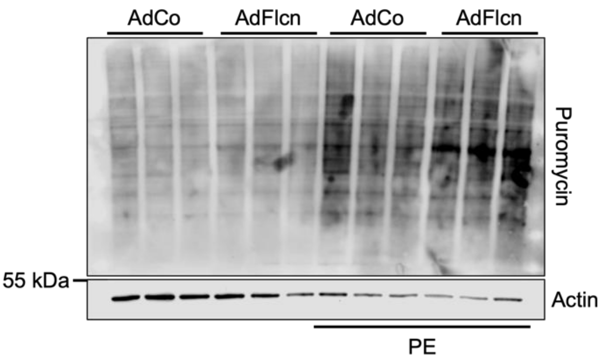

Puromycin

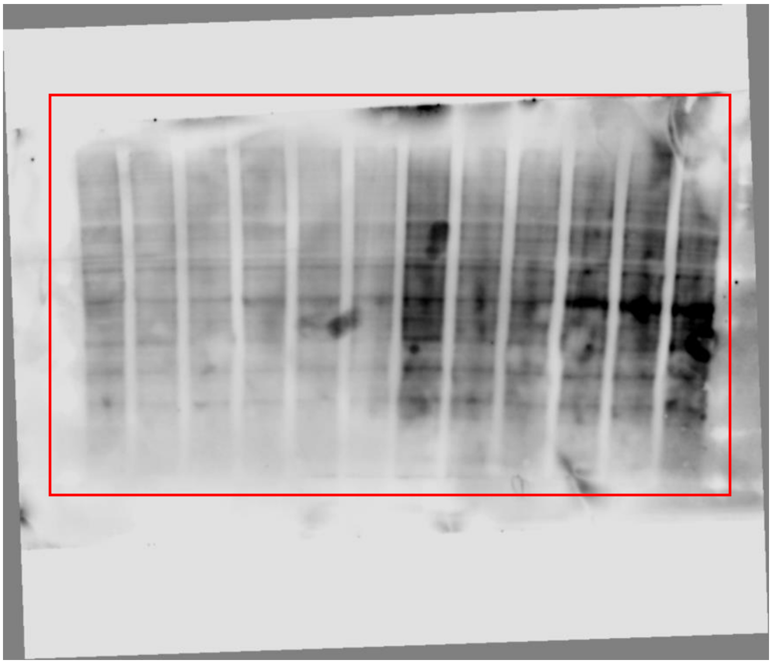

Actin

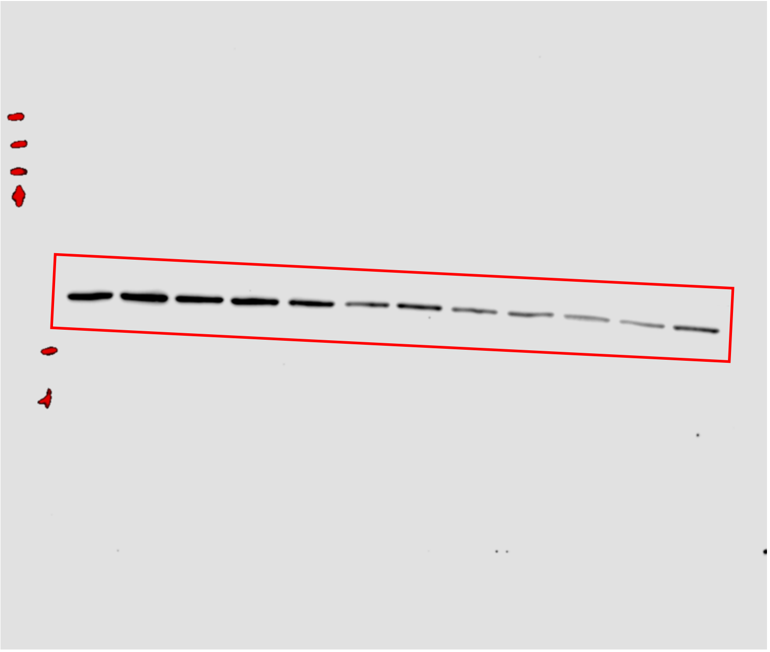

Uncropped blots – related to Figure 5i

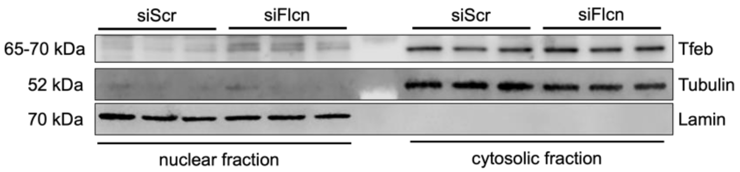

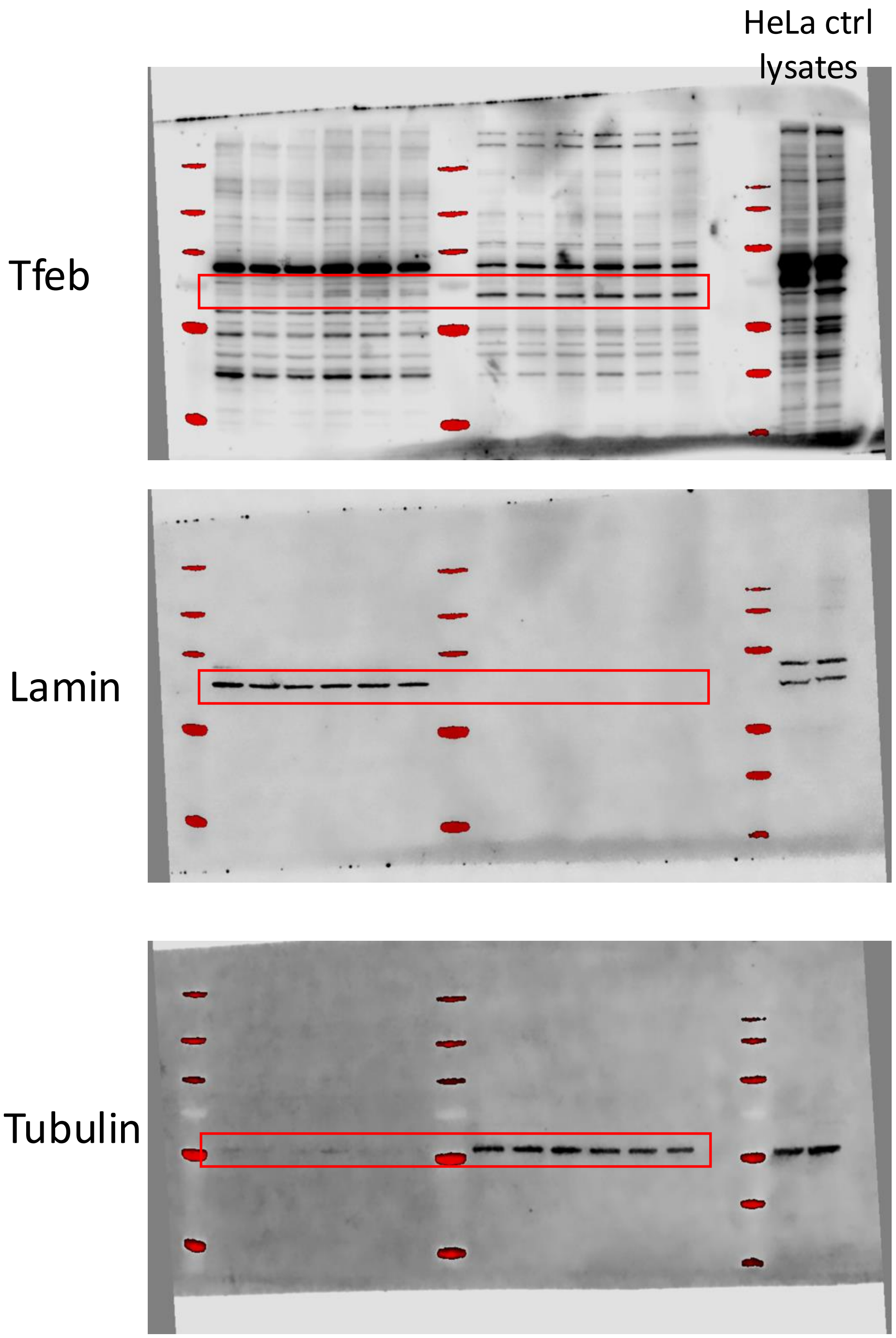

Uncropped blots – related to supplementary figure 3a

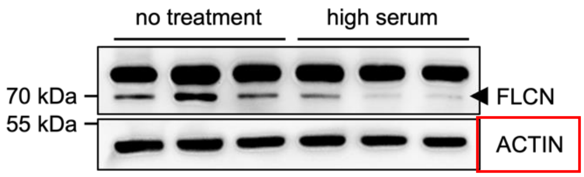

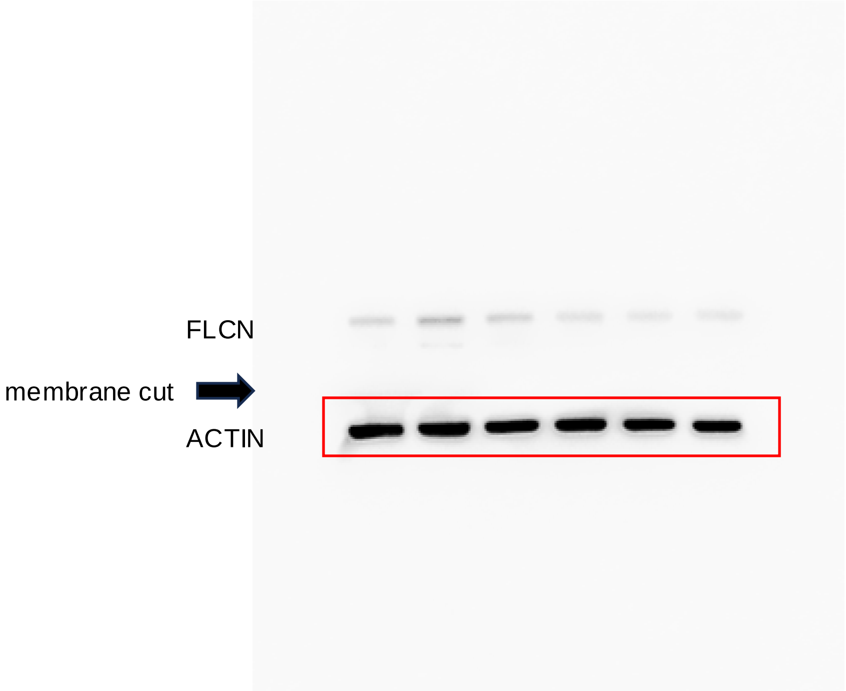

Upper part of the membrane was developed again with a longer exposure time (see next page)

Membrane overview:

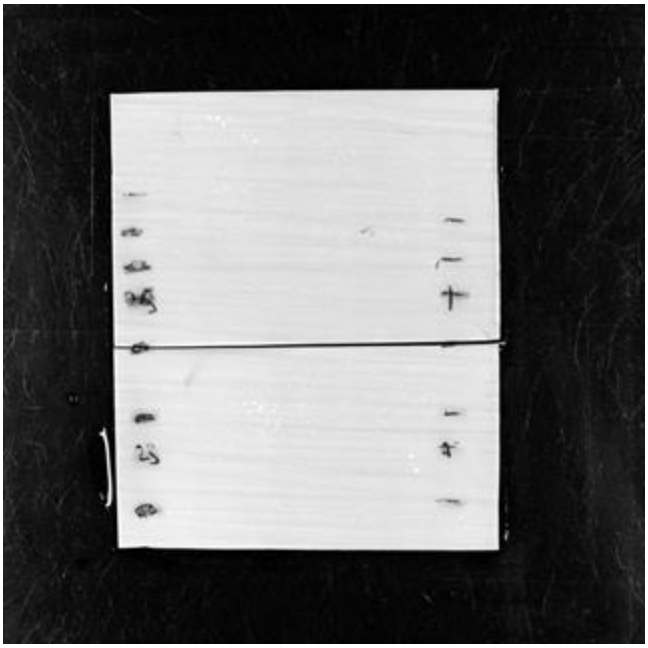

Membrane image merged with ECL image shown above:

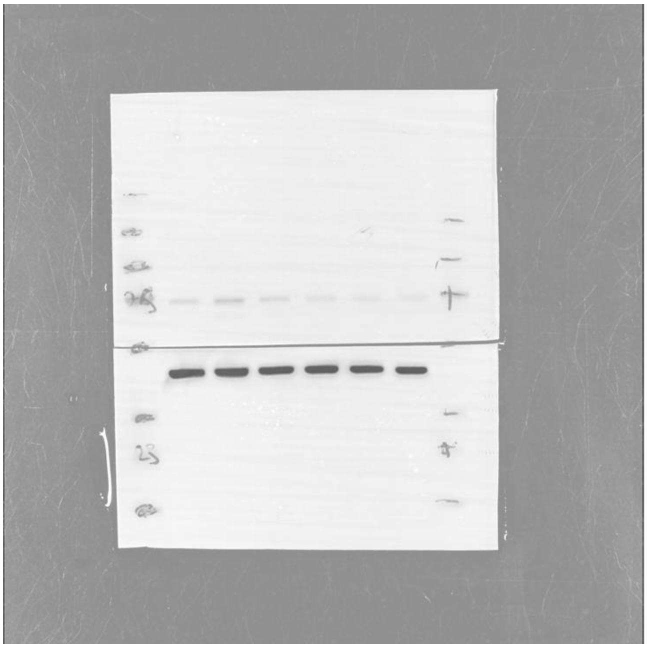

Uncropped blots – related to supplementary figure 3a

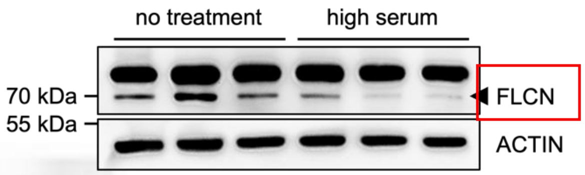

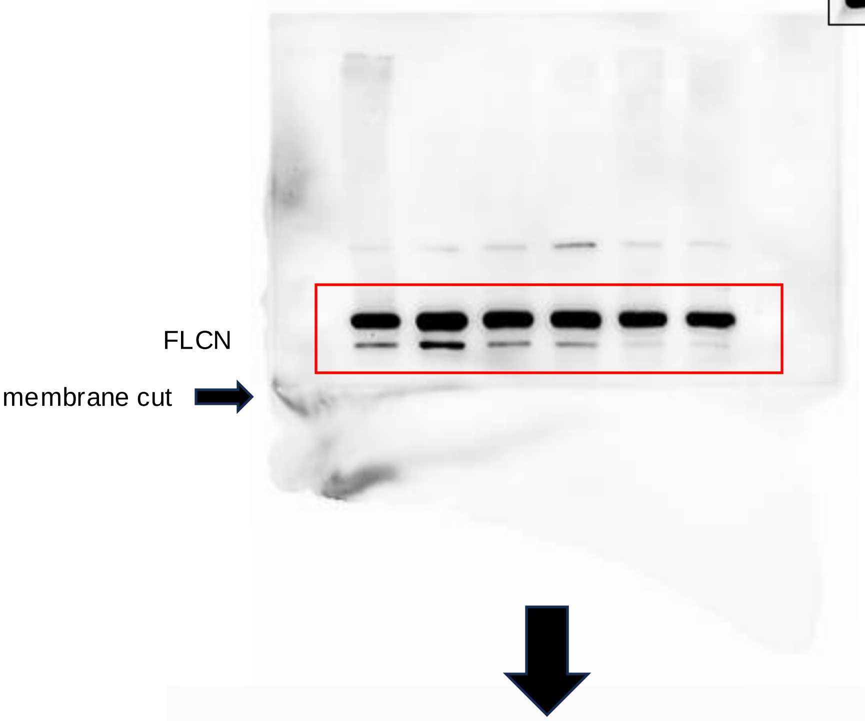

overexposed blot to show
membrane borders:

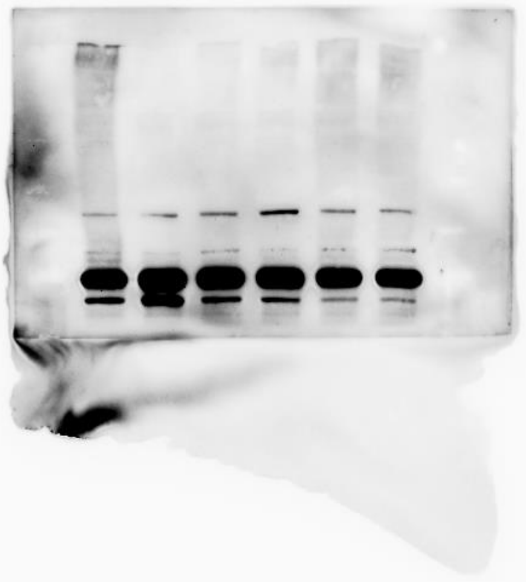

Uncropped blots – related to supplementary figure 3e

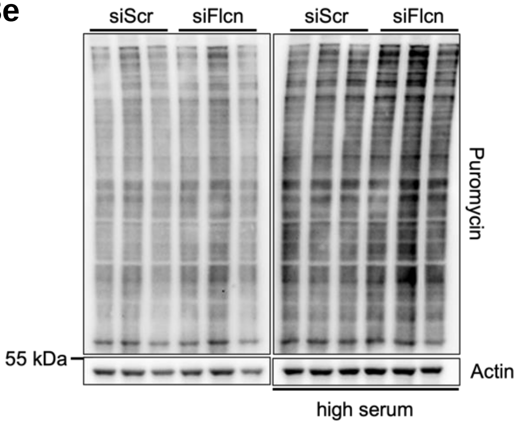

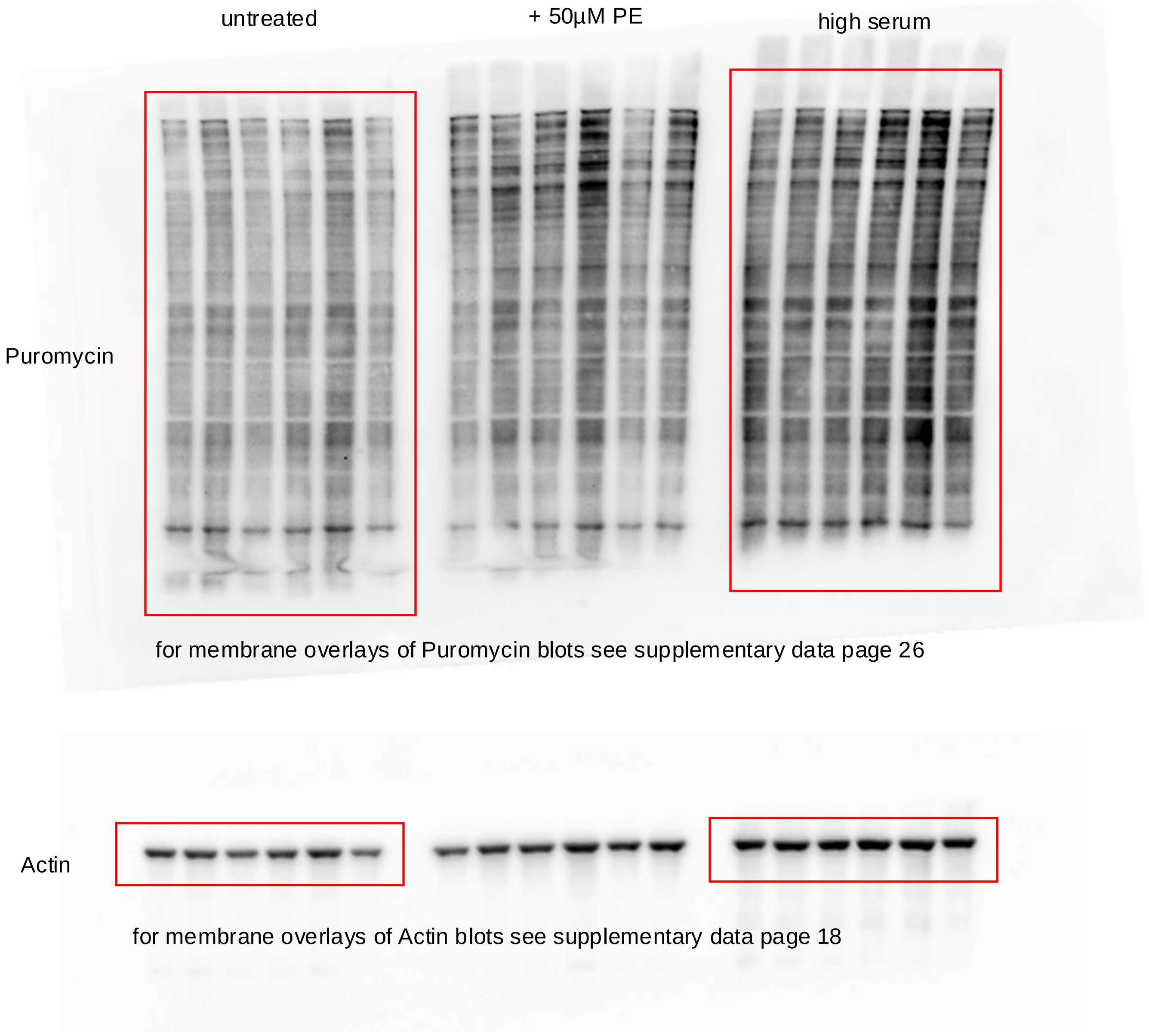

for membrane overlays of Puromycin blots see supplementary data page 26

for membrane overlays of Actin blots see supplementary data page 18

Uncropped blots – related to supplementary figure 3f

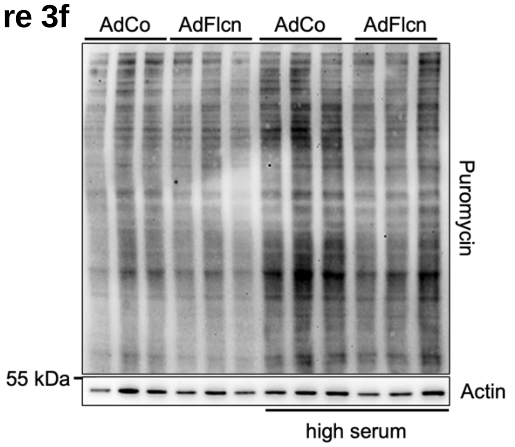

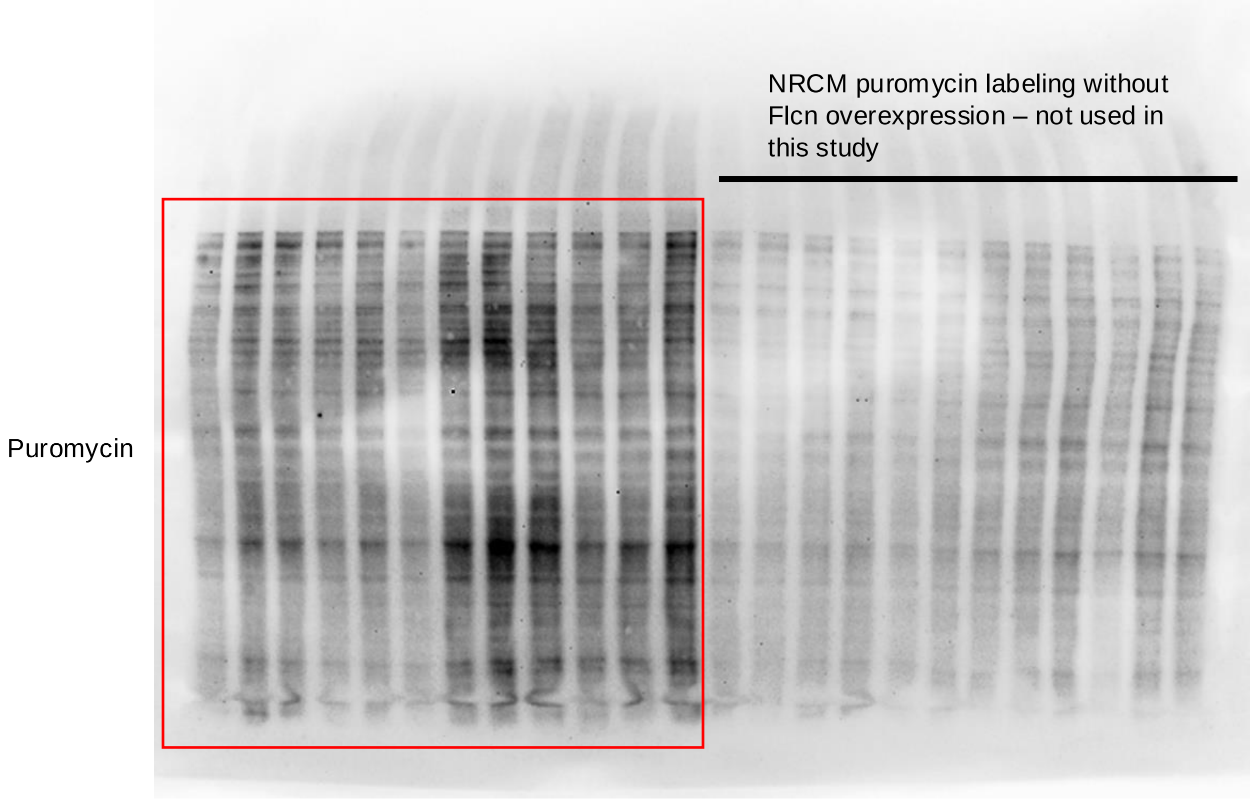

Uncropped blots – related to supplementary figure 3f

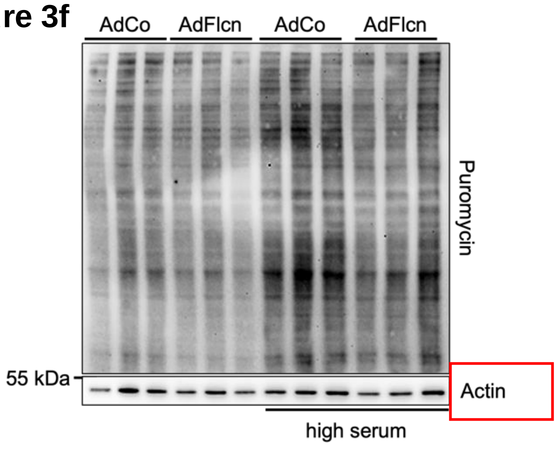

NRCM puromycin labeling without Flcn overexpression – not used in this study

membrane cut

Actin

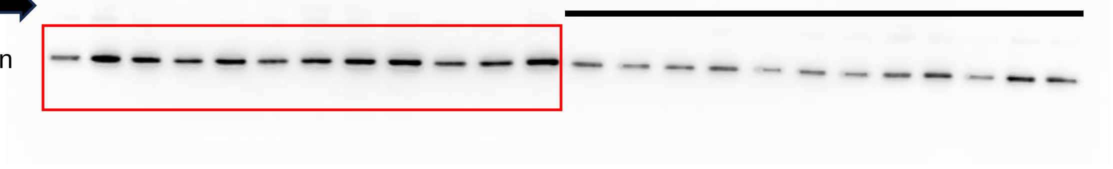

Overexposed blot to show membrane borders:

Flcn
(not shown in manuscript)

membrane cut

Actin

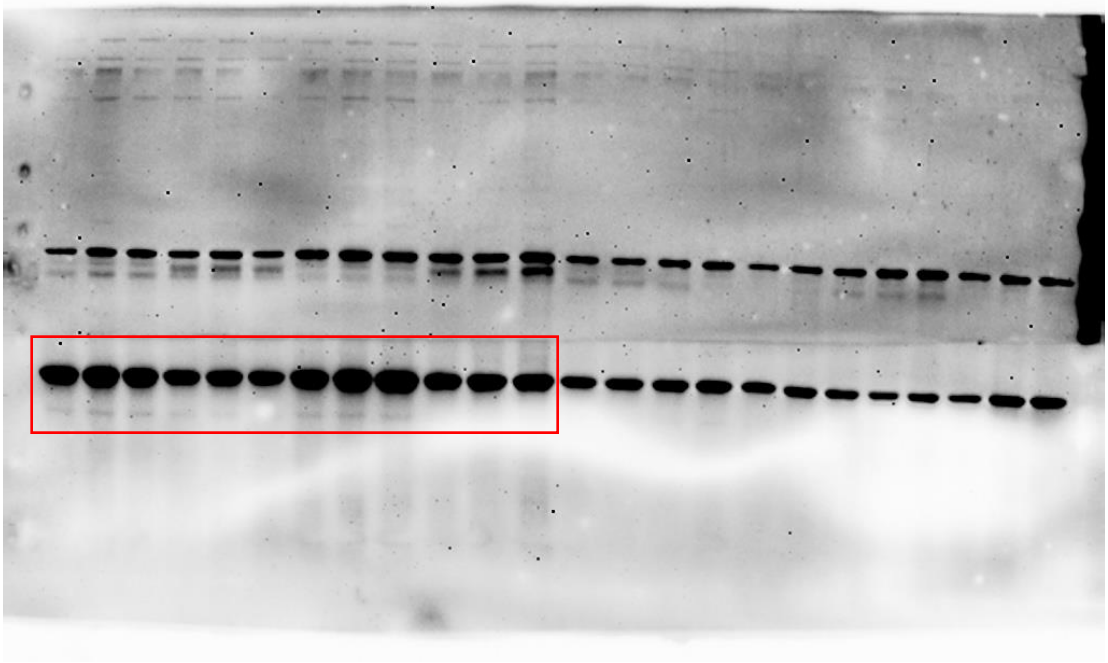

Supplement: Supplementary file 1 — Supplementary Material 1 [file 41598_2025_87107_MOESM1_ESM.pdf]
